# Stereo- and Regioselective Phyllobilane Oxidation in Leaf Homogenates of the Peace Lily (*Spathiphyllum wallisii)*: Hypothetical Endogenous Path to Yellow Chlorophyll Catabolites

**DOI:** 10.1002/chem.201404783

**Published:** 2014-11-07

**Authors:** Clemens Vergeiner, Markus Ulrich, Chengjie Li, Xiujun Liu, Thomas Müller, Bernhard Kräutler

**Affiliations:** [a] Institute of Organic Chemistry, University of Innsbruck Innrain 80/82, 6020 Innsbruck (Austria) E-mail: bernhard.kraeutler@uibk.ac.at

**Keywords:** chlorophyll, metallo-enzyme, oxidation reaction, senescence, tetrapyrrol

## Abstract

In senescent leaves, chlorophyll typically is broken down to colorless and essentially photo-inactive phyllobilanes, which are linear tetrapyrroles classified as “nonfluorescent” chlorophyll catabolites (NCCs) and dioxobilane-type NCCs (DNCCs). In homogenates of senescent leaves of the tropical evergreen *Spathiphyllum wallisii*, when left at room temperature and extracted with methanol, the major endogenous, naturally formed NCC was regio- and stereoselectively oxidized (in part) to a mixture of its 15-hydroxy and 15-methoxy derivative. In the absence of methanol in the extract, only the 15-OH-NCC was observed. The endogenous oxidation process depended upon molecular oxygen. It was inhibited by carbon monoxide, as well as by keeping the leaf homogenate and extract at low temperatures. The remarkable “oxidative activity” was inactivated by heating the homogenate for 10 min at 70 °C. Upon addition of a natural epimeric NCC (*epi*NCC) to the homogenate of senescent or green *Sp. wallisii* leaves at room temperature, the exogenous *epi*NCC was oxidized regio- and stereoselectively to 15-OH-*epi*NCC and 15-OMe-*epi*NCC. The identical two oxidized *epi*NCCs were also obtained as products of the oxidation of *epi*NCC with dicyanodichlorobenzoquinone (DDQ). Water elimination from 15-OH-*epi*NCC occurred readily and gave a known “yellow” chlorophyll catabolite (YCC). The endogenous oxidation process, described here, may represent the elusive natural path from the colorless NCCs to yellow and pink coloured phyllobilins, which were found in (extracts of) some senescent leaves.

## Introduction

**Scheme 1:**
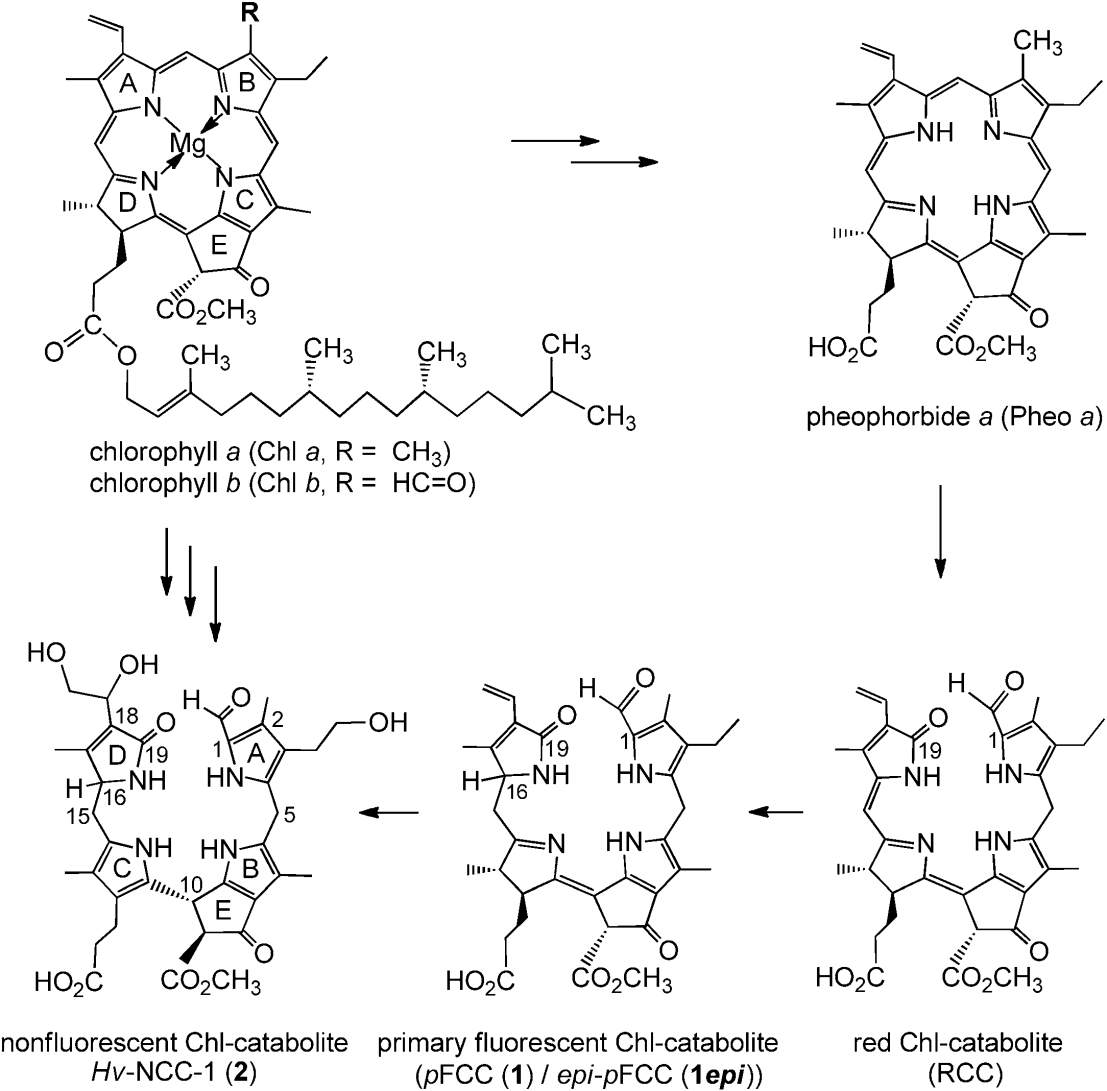
Structural outline of chlorophyll breakdown in senescent leaves. Chlorophylls *a*&*b* (Chl *a*&Chl *b*) are first degraded to pheophorbide *a* (Pheo *a*). Ring opening of the chlorin macrocycle of Pheo *a* provides an enzyme bound red chlorophyll catabolite (RCC), which is subsequently reduced to the “primary” fluorescent chlorophyll catabolite (*p*FCC, 1) or its 16-epimer (*epi-p*FCC, 1*epi*). *p*FCCs (1/1*epi*) undergo further modifications and isomerization to “non-fluorescent” chlorophyll catabolites (NCCs), such as *Hv-*NCC-1 (2), first detected in primary leaves of barley (*Hordeum vulgare*).[3b, 4]

Breakdown of chlorophyll (Chl) accompanies the fascinating color changes in de-greening leaves and ripening fruit.[1] It is the source of a group of abundant Chl-derived linear tetrapyrroles,[2] now generically named phyllobilins.[3] In senescent leaves and ripening fruit, mostly colorless chlorophyll catabolites (CCs) accumulate, such as the “non-fluorescent” CCs (NCCs), which were the first non-green tetrapyrrolic degradation products of Chl to be identified.[4] In senescent leaves of a variety of higher plants, the rapidly formed colorless NCCs[5] were typically observed as the “final” products of Chl breakdown. In other leaves, mostly colorless “dioxobilin-type” NCCs (DNCCs) were found,[6] formal (but not genuine) deformylation products of NCCs.[6b] The typical accumulation of colorless and photo-inactive phyllobilins was interpreted as the result of a controlled Chl detoxification process (see Scheme 1).[5] The common precursor of NCCs and DNCCs are the “primary” fluorescent CCs *p*FCC**/***epi-p*FCC. These (C16)-epimeric *p*FCCs are mostly short lived and isomerize rapidly in (weakly acidic) aqueous medium to the corresponding NCCs, whose newly formed saturated C10 was assigned *R* configuration, based on the mechanistic analysis of their highly stereoselective isomerization process.[7] However, in yellow senescent leaves of banana plants (*Musa acuminata*),[8] and of the Peace Lily (*Spathiphyllum wallisii*),[9] persistent, “hyper-modified fluorescent” CCs (*hm*FCCs) accumulated, whereas NCCs either appeared to be completely absent (banana leaves[8]), or they were apparently found only in small amounts (Peace Lily leaves[9]). Persistent *hm*FCCs are sources of remarkable “fluorescence” signals[10] and effective sensitizers of the endogenous, light-induced formation of singlet oxygen (^1^O_2_),[11] and may have further biological roles in plants.[12]

**Scheme 2:**
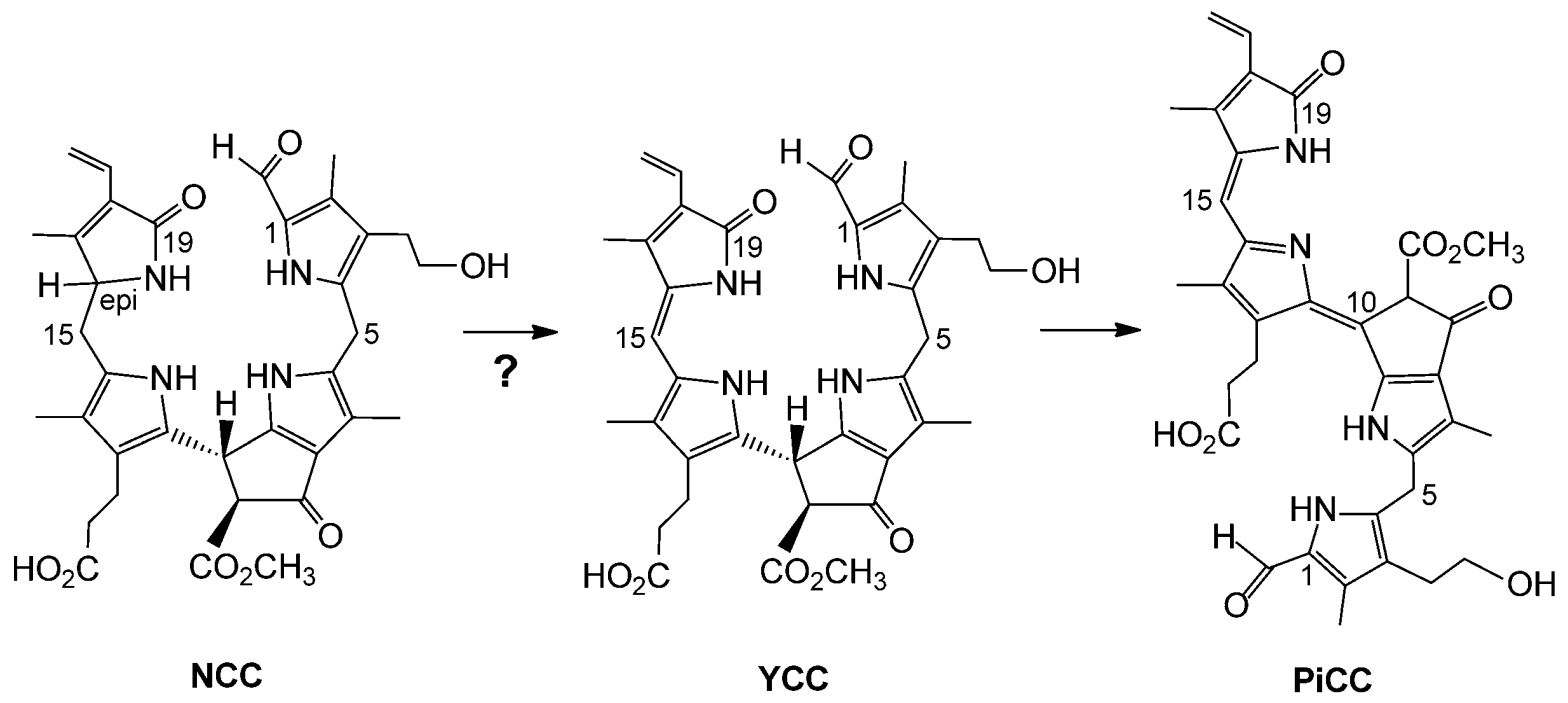
In senescent leaves of *C. japonicum* the yellow CC (YCC) 7 and pink CC (PiCC) 8 were observed as the presumed products of the NCC 4*epi*.[13b]

The recent discovery of yellow Chl catabolites (YCCs) and pink chlorophyll catabolites (PiCCs) (see Scheme 2) in senescent leaves of the Katsura tree (*Cercidiphyllum japonicum*)[13] and of other deciduous plants[14] indicated further oxidative transformations of NCCs, which are noted antioxidants.[15] However, a pathway for the suggested endogenous formation of YCCs and PiCCs has remained elusive.[3b, 5] Here we report on the selective oxidation of natural NCCs in homogenized *Sp. wallisii* leaves. Via this process, 15-hydroxy- and 15-methoxy-NCCs are formed with high stereo- and regioselectivity. Such “oxidized” NCCs are precursors for a known YCC, thus providing an endogenous oxidative path from NCCs to YCCs that may be active in leaves.

## Results and Discussion

### i) Analysis of Chl-catabolites in extracts of senescent *Spathiphyllum wallisii* leaves—Formation of oxidized phyllobilanes

**Figure 1:**
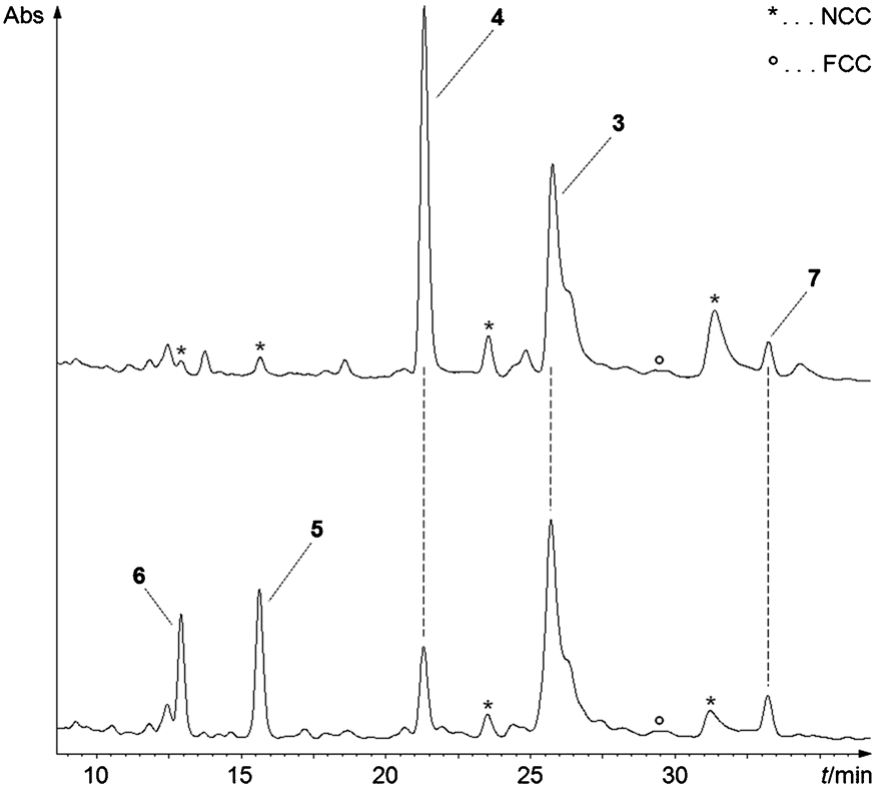
HPLC analyses of Chl catabolites in extracts of yellow senescent *Spathiphyllum wallisii* leaves. Top: extract was prepared from fresh leaf material frozen with liquid nitrogen, and shows NCC 4 and FCC 3 as main catabolites. Bottom: extract prepared at ambient temperature.[9] The HPL-chromatograms were recorded with detection at 320 nm. Minor fractions tentatively classified as fluorescent (FCCs) or non-fluorescent chlorophyll catabolites (NCCs) are labelled by compound type only (*=NCC, ○=FCC).

Preparation of an extract of senescent *Sp. wallisii* leaves at ambient temperature and analysis by HPLC indicated the presence of several (minor) fractions with UV/Vis-absorption characteristics typical of NCCs, as well as of a major “fluorescent” CC (FCC), named *Sw*-FCC-62 (**3**).[9] The least polar among the NCCs, named *Sw*-NCC-58 (**4**),[9] was characterized as a 1-formyl-3^2^-hydroxy-19-oxo-16*n*-16,19-dihydrophyllobilane (for nomenclature and atom numbering see Experimental Section, Figure 7). In addition, several other presumed CCs were indicated, such as the polar NCCs **5** and **6**, and the “yellow” CC (YCC) **7**. In contrast, when such an extract was prepared from fresh leaf material frozen with liquid nitrogen, FCC **3** and NCC **4** represented the major fractions of Chl catabolites, whereas only traces of **5** and **6** were present (see Figure 1).

**Figure 2:**
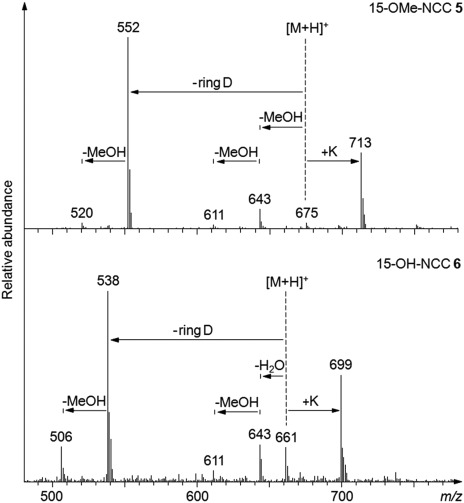
ESI-Mass spectra of oxidized NCCs 5 and 6 (see Experimental Part for details)

Samples of the three NCC fractions were isolated by semi-preparative HPLC from an extract of 5 g (wet weight) of yellow-greenish *Sp. wallisii* leaves. The least polar NCC fraction from such leaves was identified as the known *Sw*-NCC-58 (**4**).[8] A sample of 370 nmol (250 μg) of the slightly more polar fraction of the NCC *Sw*-NCC-51 (**5**) was also isolated, whose UV and CD spectra (see Supporting Information, Figure S1) were similar to those of **4**.[9] When analyzed by positive ion ESI-mass spectrometry, pseudo-molecular ions [*M*+K]^+^ and [*M*+H]^+^ were found at *m*/*z* 713.3 and *m*/*z* 675.4, respectively, consistent with a molecular formula of **5** of C_36_H_42_N_4_O_9_ (indicating the formal addition of a CH_2_O unit to **4**). Fragments at *m*/*z* 643.3 and 611.2 showed loss of one and of two units of methanol, and a fragment at *m*/*z* 552.2 suggested loss of ring D (C_7_H_9_NO) (see Figure 2).

**Scheme 3:**
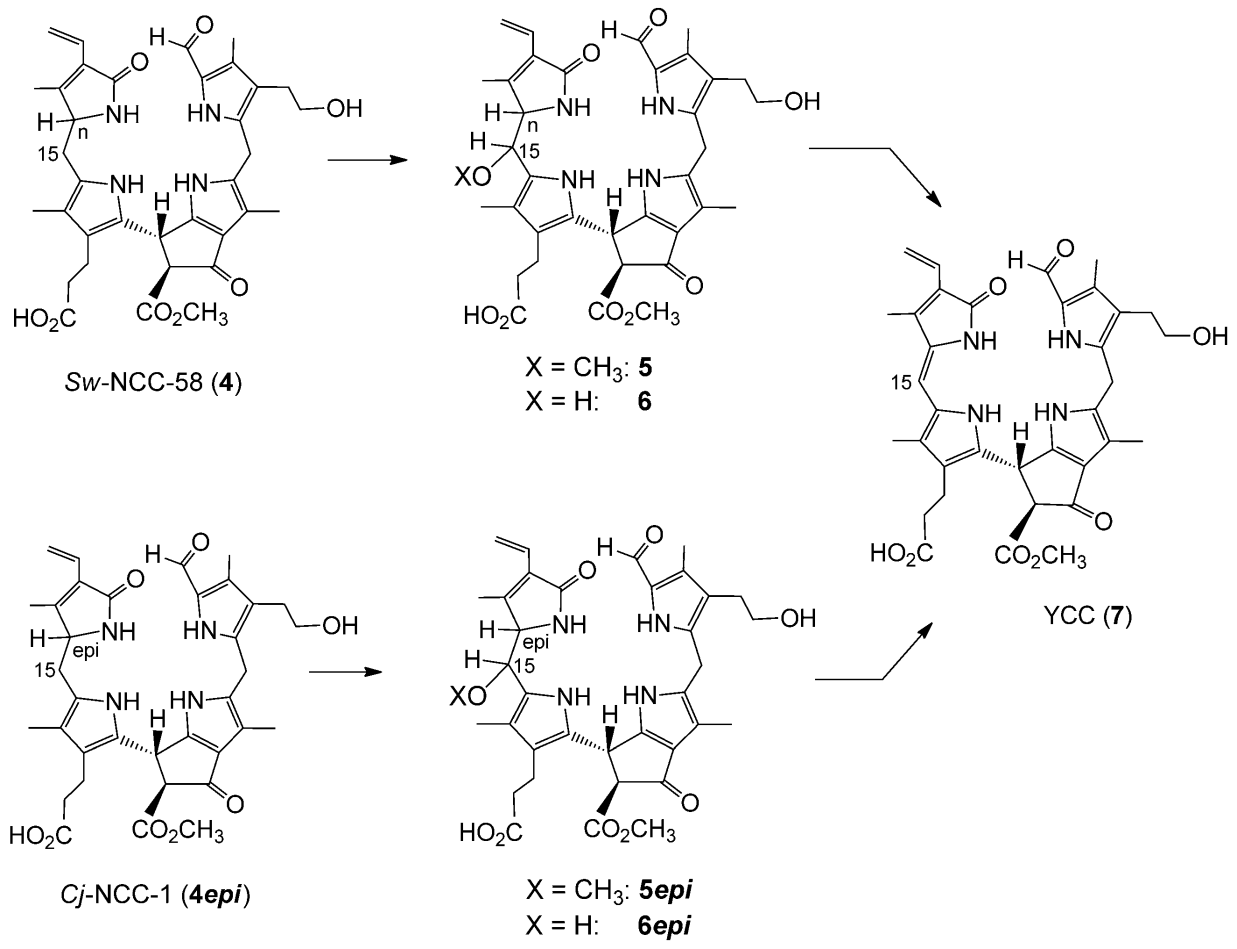
Schematic path of the formation of oxidized NCCs 5 and 6 from 4 (resp. 5*epi* and 6*epi* from 4*epi*) and their further conversion to YCC 7.

The ^1^H NMR spectrum of the NCC **5** in CD_3_OD at 10 °C displayed the set of the characteristic signals of a 1-formyl-19-oxo-phyllobilane; among them signals at low field due to a formyl and a vinyl group, four singlets of methyl groups at high field, as well as singlets at 3.12 ppm (methoxy group) and at 3.76 ppm (ester methyl group). A doublet at 3.78 ppm (assigned to HC15) coupled with another doublet at 4.10 ppm (HC16). Thus, C15 of **5** was indicated to carry a new substituent (in **4** H_2_C15 gave rise to a double doublet at 2.51 and 2.80 ppm). ^1^H,^1^H-NOE correlations of HC15 with a singlet at 3.12 ppm (H_3_CO15^2^) and with singlets at 1.95 ppm (H_3_C13^1^) and at 1.06 ppm (H_3_C17^1^) helped to secure this assignment. In sum, the signals of 35 non-exchangeable protons of NCC **5** could be assigned. Detailed information from multidimensional NMR spectra (^1^H,^1^H-COSY and ROESY spectra, see Supporting Information, Figure S2) allowed the delineation of the constitution of the NCC **5** as that of an unprecedented 1-formyl-3^2^-hydroxy-15-methoxy-19-oxo-16,19-dihydrophyllobilane, also identifying **5** as “15-OMe-*Sw*-NCC-58” (see Scheme 3).

The slightly more polar, less abundant fraction of the NCC **6** was also identified provisionally as an NCC related to **4**, based on its UV and mass spectra (see Figure 2 and Supporting Information Figure S1). The positive-ion ESI-mass spectrum of **6** exhibited pseudo molecular ions [*M*+K]^+^ and [*M*+H]^+^ at *m*/*z* 699.3 and *m*/*z* 661.3, consistent with the molecular formula C_35_H_40_N_4_O_9_ (indicating the formal addition of an O atom to **4**). Fragment ions at *m*/*z* 643.3 and 611.3 indicated loss of water and, in addition, of methanol, and efficient loss of ring D was suggested by an intense signal at *m*/*z* 538.2 (see Figure 2). This fragmentation pattern suggested the NCC **6** to be the 15-OH analogue of **5** (see Scheme 3).

Significant amounts of isomers of **5** and **6** were absent in the extracts of senescent *Sp. wallisii* leaves, suggesting the hypothetical paths of the formation of **5** and **6** from **4** to be very stereo- and regioselective, and possibly, to involve enzyme-catalyzed processes. As shown below, in the absence of added methanol, only the formation of 15-hydroxy-NCC **6** may actually occur in senescent *Sp. wallisii* leaves. Remarkably, the C15 hydroxylation would involve an NCC, that is **4**, as substrate. In contrast, peripheral hydroxy groups that are typical of NCCs, were suggested earlier to be introduced at the (preceding) stage of FCCs.[5]

In addition to the polar NCCs **5** and **6**, a less polar yellow fraction was found in the extract, classified as a YCC on the basis of its UV/Vis spectrum with a prominent absorption at 426 nm (see Figure S3). When analyzed by ESI-MS, the pseudo-molecular ion [*M*+H]^+^ of this YCC occurred at *m*/*z* 643.2, indicating a molecular formula of C_35_H_38_N_4_O_8_. HPLC characteristics, UV/Vis and mass spectra suggest its identity with the yellow catabolite *Cj*-YCC (**7**), previously found in naturally de-greened leaves of *Cercidiphyllum japonicum*.[13a] YCC **7** is a presumed oxidation product of **4**, and is readily formed from a 15-OH-NCC by elimination of water from the 15- and 16-positions (see below).

### ii) Homogenates of *Sp. wallisii* leaves oxidize an added NCC stereo- and regioselectively

**Figure 3:**
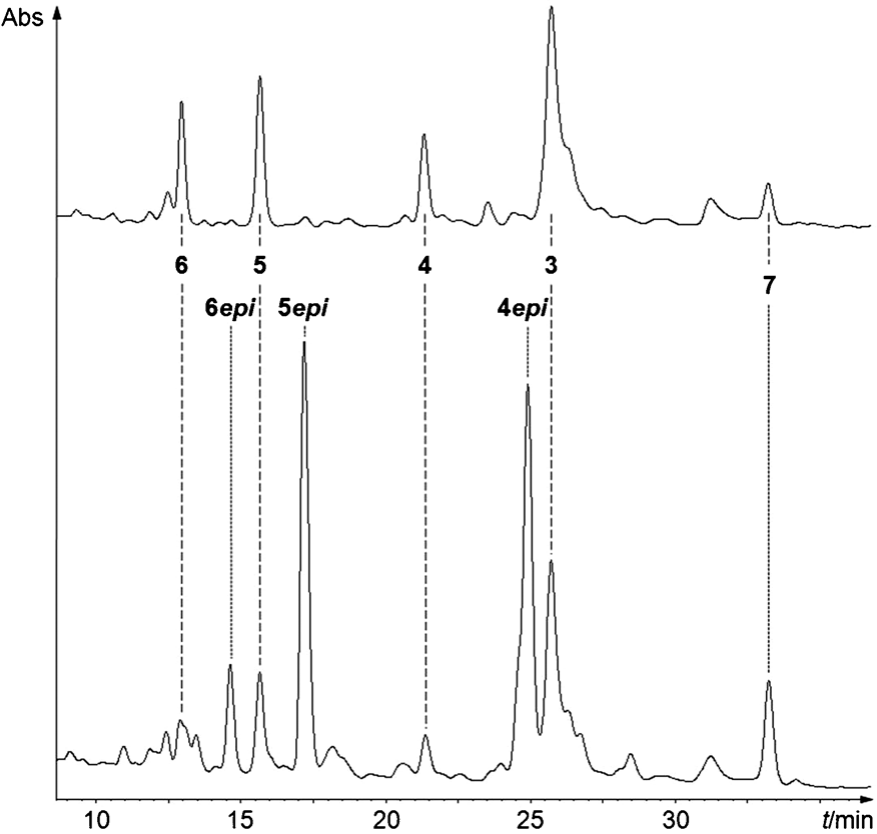
HPL-chromatograms of extracts of yellow senescent *Sp. wallisii* leaves (detection at 320 nm). Top: analysis of an extract prepared at ambient temperature. Bottom: analysis of the analogous extract, produced with addition of about 0.1 mg of 4*epi* to the sample of senescent *Sp. wallisii* leaves, followed by usual work-up. Note: the oxidized NCCs obtained with the endogenous NCC 4 and the added NCC 4*epi* show different retention times, whereas only a single YCC 7 is produced.

In order to find evidence for the suggested process of a regio- and stereoselective oxidation of NCC **4** to the more polar NCCs **5** and **6** in homogenates of senescent *Sp. wallisii* leaves, the epimeric NCC **4*epi*** (from senescent leaves of *Cercidiphyllum japonicum*)[16] was incubated with freshly ground *Sp. wallisii* leaves, either senescent or still green. In both of these latter cases, HPLC-analysis showed the formation of more polar NCCs, identified as **5*epi*** and **6*epi*** (see Scheme 3 and Figure 3).

For the preparation of these polar NCCs from **4*epi*** a homogenate of a green *Sp. wallisii* leaf was used, to simplify the isolation procedure. In this experiment, a sample of 12.5 mg (19.4 μmol) of **4*epi***, suspended in water, was thus mixed with freshly ground leaf material obtained from 25 cm^2^ of green *Sp. wallisii* leaves, and the slurry was stirred for 6 h at ambient temperature in the dark, whereupon MeOH was added for work-up. Analysis of the reaction mixture by HPLC indicated the presence of three fractions with UV/Vis spectra of NCCs, and one fraction with the spectrum of a YCC (see Supporting Information, Figure S3). Quantitative UV/Vis-spectroscopic analysis of these product fractions indicated formation of 10.7 μmol (55 % overall) of the oxidation products **5*epi***, **6*epi*** and **7**, and recovery of 14 % of **4*epi*** (which was added initially). Purification of the raw product by preparative HPLC furnished two NCC fractions and a sample of YCC **7**, besides about 2.7 μmol of recovered **4*epi***. From the main NCC fraction, a slightly yellow powder of **6*epi*** (1.9 mg, 2.9 μmol) was obtained, after further work-up and isolation, as well as smaller samples of **5*epi*** and of **7**).

**Scheme 4:**
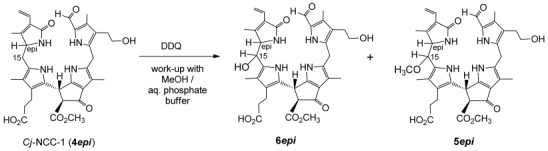
Oxidation of 4*epi* with dicyano-dichloro-benzoquinone (DDQ) and direct work-up with addition of methanol and water.

A polar fraction from this experiment had UV- and CD-spectral properties of a typical NCC (see Supporting Information, Figure S1). The positive-ion ESI-mass spectrum of this NCC (**6*epi***) exhibited its [*M*+H]^+^ ion at *m*/*z* 661.1 (see Supporting Information, Figure S4) consistent with a molecular formula of C_35_H_40_N_4_O_9_ and indicating **6*epi*** to be an isomer of **6**. The ^1^H NMR spectrum of a solution of the NCC **6*epi*** in CD_3_OD at 10 °C (see Supporting Information, Figure S5) showed the set of characteristic signals of the tetrapyrrole core of a 1-formyl-19-oxo-phyllobilane, for example, signals at low field from a formyl and a vinyl group and four singlets of methyl groups at high field. In the intermediate field, only one sharp singlet at 3.74 ppm (of a methyl ester group) was observed. The strong downfield shift of HC15 (to 4.38 ppm) as part of an AB system at 4.24/4.38 ppm (*J*=7.5) was consistent with the presence of a de-shielding substituent at C15. ^1^H,^1^H-NOE correlations of HC15 with methyl group singlets at 2.00 ppm (H_3_C13^1^) and at 1.52 ppm (H_3_C17^1^) helped to put this assignment on a safe ground. In sum, the signals of 32 non-exchangeable protons of this NCC could be assigned. Detailed information from multidimensional NMR spectra (^1^H,^1^H-COSY and ROESY spectra, see Supporting Information, Figure S5) allowed the delineation of the constitution of this “polar” 15-OH-NCC as a 1-formyl-3^2^,15-dihydroxy-19-oxo-16*epi*-16,19-dihydrophyllobilane. Furthermore, the sample of **6*epi*** had the same HPL-chromatographic and NMR-spectroscopic properties as a 15-HO-NCC that was obtained from chemical oxidation of **4*epi*** with dicyano-dichlorobenzoquinone (DDQ), (see below and Schemes 3 and 4).

The slightly less polar NCC **5*epi*** (with UV and CD spectra of a typical NCC, see Supporting Information, Figure S1) showed a pseudo molecular ion [*M*+H]^+^ at *m*/*z* 675.0 (ESI-MS spectrum, see Supporting Information, Figure S4), consistent with a molecular formula of **5*epi*** of C_36_H_42_N_4_O_9_. This indicated formal addition of a CH_2_O unit to **4*epi***. The sample was identified by HPLC-co-injection with a 15-MeO-NCC, obtained from chemical oxidation of **4*epi*** with DDQ, and which was characterized as a 1-formyl-3^2^-hydroxy-15-methoxy-19-oxo-16*epi*-16,19-dihydrophyllobilane (see below and Schemes 3 and 4).

In the green leaf homogenate, **4*epi*** was thus oxidized, in part, and in the methanol extract a 15-hydroxy-*epi*NCC and a 15-methoxy-*epi*NCC was found. When using a ground senescent *Sp. wallisii* leaf for transformation of **4*epi***, the endogenous NCC **4** was also converted in parallel, furnishing additional small amounts of the NCCs **5** and **6**, both of which were slightly more polar than their counterparts from **4*epi*** (see Figure 3). Significant amounts of other isomers of **5*epi*** and **6*epi*** were absent in the experiments with senescent *Sp. wallisii* leaves, suggesting formation of **5*epi*** and **6*epi*** from **4*epi*** to be very stereo- and regioselective.

The less polar yellow fraction found in *Sp. wallisii* leaf extracts, and identified as YCC **7**, was also observed when **4*epi*** was oxidized in the extract (see Figure 3). Obviously, formation of **7** (as common product) from oxidation of **4** or of **4*epi*** at their respective 15-position, and elimination of water, entailed the loss of asymmetry at C16, which gave rise to the epimeric relationship between **4** and **4*epi***. This finding was thus consistent with our earlier assignment of **4** (from *Sp. wallisii*)[9] as C16-epimer of **4*epi*** (from *Cercidiphyllum japonicum*).[16]

### iii) Preparation of 5*epi* and of 6*epi* via oxidation of 4*epi* with 2,3-dichloro-5,6-dicyano-1,4-benzoquinone (DDQ)

Oxidation with DDQ was used earlier for the synthesis of YCC **7**.[13a] Using the published procedure for the oxidation of **4*epi***, followed by a different work-up, a mixture of the two oxidized NCCs, **5*epi*** and **6*epi***, could be prepared. A solution of 201.4 mg (312.4 μmol) of **4*epi*** in acetone was treated with 78 mg (1.1 equiv) of DDQ.[13a] The rust-colored raw intermediary product was dissolved in MeOH at room temperature and phosphate buffer (pH 7) was added. The brown solution was applied to an RP-18 column, to be separated by MPLC: three fractions containing NCCs (from UV/Vis spectra) and one YCC-fraction were collected and the corresponding purified tetrapyrroles were isolated (see Schemes 3 and 4).

The first, most polar NCC fraction (18.7 mg, 28.3 μmol, 9.1 %) was classified on the basis of its UV spectrum, which was similar to the one of **4*epi***, the starting material. A positive ion ESI mass spectrum of this polar NCC displayed the pseudo-molecular ion [*M*+K]^+^ at *m*/*z* 699.4, and the corresponding sodium adduct [*M*+Na]^+^ at *m*/*z* 683.4. This was consistent with a molecular formula of C_35_H_40_N_4_O_9_, as found in the HO-NCC **6*epi***, suggesting the formal addition of an oxygen atom to **4*epi***. A fragment at *m*/*z* 560.3 (indicating loss of ring D=C_7_H_9_NO) from the sodium adduct, was also found (as in the mass spectrum of **6*epi***). Identification of 15-OH-*epi*NCC from the DDQ-oxidation with the 1-formyl-3^2^,15-dihydroxy-19-oxo-16*epi*-16,19-dihydro-phyllobilane **6*epi*** from the “biological” oxidation in *Spathiphyllum* leaves was secured by complete spectral comparison (UV, ESI-MS, ^1^H and ^13^C NMR), as well as by HPLC (see above, Scheme 3 and below).

A slightly less polar fraction was isolated as 42.6 mg (63.1 μmol, 20.2 % yield) of a slightly yellowish powder, and was also classified as an NCC based on its UV-spectrum. Analyses by positive ion ESI mass spectrometry showed a pseudo-molecular ion [*M*+Na]^+^ at *m*/*z* 697.4, consistent with its molecular formula as C_36_H_42_N_4_O_9_ (and indicating the formal addition of a CH_2_O unit to **4*epi***). The ^1^H NMR spectrum of this NCC in CD_3_OD at 25 °C (see Supporting Information, Figure S6) showed the set of typical signals of a 1-formyl-19-oxo-phyllobilane. Characteristic signals of a formyl and a vinyl group occurred at low field, four singlets of methyl groups at high field, as well as a (methyl ester) singlet at 3.77 ppm, were observed. A doublet at 4.28 ppm (assigned to HC16) showed coupling (*J*=9.1 Hz) to another doublet at 3.88 ppm, which was assigned to the single proton at C15. Besides the correlations in ^1^H,^1^H-ROESY experiments of HC15 with two singlets, at 2.01 (H_3_C13^1^) and at 1.42 ppm (H_3_C17^1^), a further correlation to a methyl group singlet at 3.07 was observed. This singlet was assigned to the new methoxy group attached at C15 (see also identification of **5**, above). In brief, the signals of overall 35 non-exchangeable protons of this NCC could be identified. 2D NMR experiments (^1^H,^1^H-COSY, ^1^H,^1^H-ROESY, ^1^H,^13^C-HSQC and ^1^H,^13^C-HMBC, see Supporting Information, Figure S6), and additional NMR experiments in CD_3_CN/CHCl_3_ solution, helped assign this 15-OMe-*epi*NCC the structure of a 1-formyl-3^2^-hydroxy-15-methoxy-19-oxo-16*epi*-16,19-dihydrophyllobilane, and further HPLC -analysis (see below), and allowed its identification with **5*epi***.

The occurrence, in parallel, of the stereo- and regioselective formation of the 15-OH- and 15-OMe-*epi*NCCs from “chemical” and “biological” oxidations of **4*epi***, as well as the similarity of the ^1^H- and ^13^C NMR chemical shift data of the “western” moieties of the oxidation products, suggest a common relative configuration of their stereocenters at C15 and at C16. Thus, **6*epi*** would be the 15-OH analogue of **5*epi*** (and vice versa).

The least polar NCC (42.8 mg, 21.3 %) was identified as re-isolated starting material (**4*epi***), based on the HPLC-retention time, its UV spectrum and a pseudo molecular ion at *m*/*z* 645.2 [*M*+H]^+^ indicating the molecular formula to be C_35_H_40_N_4_O_8_.

Besides the two new NCCs, characterized as **5*epi*** and **6*epi***, a less polar yellow fraction was isolated (60.5 mg, 31 %), identified as YCC **7**:[13a] Its UV/Vis spectrum exhibited a prominent absorption maximum at 426 nm; its pseudo-molecular ion [*M*+H]^+^ at *m*/*z* 643.2 in the ESI mass spectrum indicated the molecular formula of C_35_H_38_N_4_O_8_. YCC **7** is a formal dehydrogenation product of **4*epi***, which presumably was formed by elimination of methanol or water from the C15 and C16 positions of **5*epi*** and **6*epi***, respectively (see below).

### iv) Identification of samples of 5*epi* and of 6*epi* from oxidations of 4*epi* with an extract of *Sp. wallisii* leaves and by DDQ

Samples of **5*epi*** and **6*epi*** obtained from oxidation of **4*epi*** with homogenates of *Sp. wallisii* leaves, showed the same HPLC-elution properties as those of the 15-OMe-*epi*NCC and of the 15-OH-*epi*NCC (respectively), obtained from treatment of **4*epi*** with DDQ. The two samples obtained from each of these oxidations of **4*epi*** were identified in HPLC co-injection experiments (see Figure S7). Thus, two NCC samples were obtained from both oxidations of **4*epi*** that were identical pair wise (also with respect to the selectively generated new stereocenter at C15), and that, both, represented **5*epi*** and **6*epi***.

**Scheme 5:**
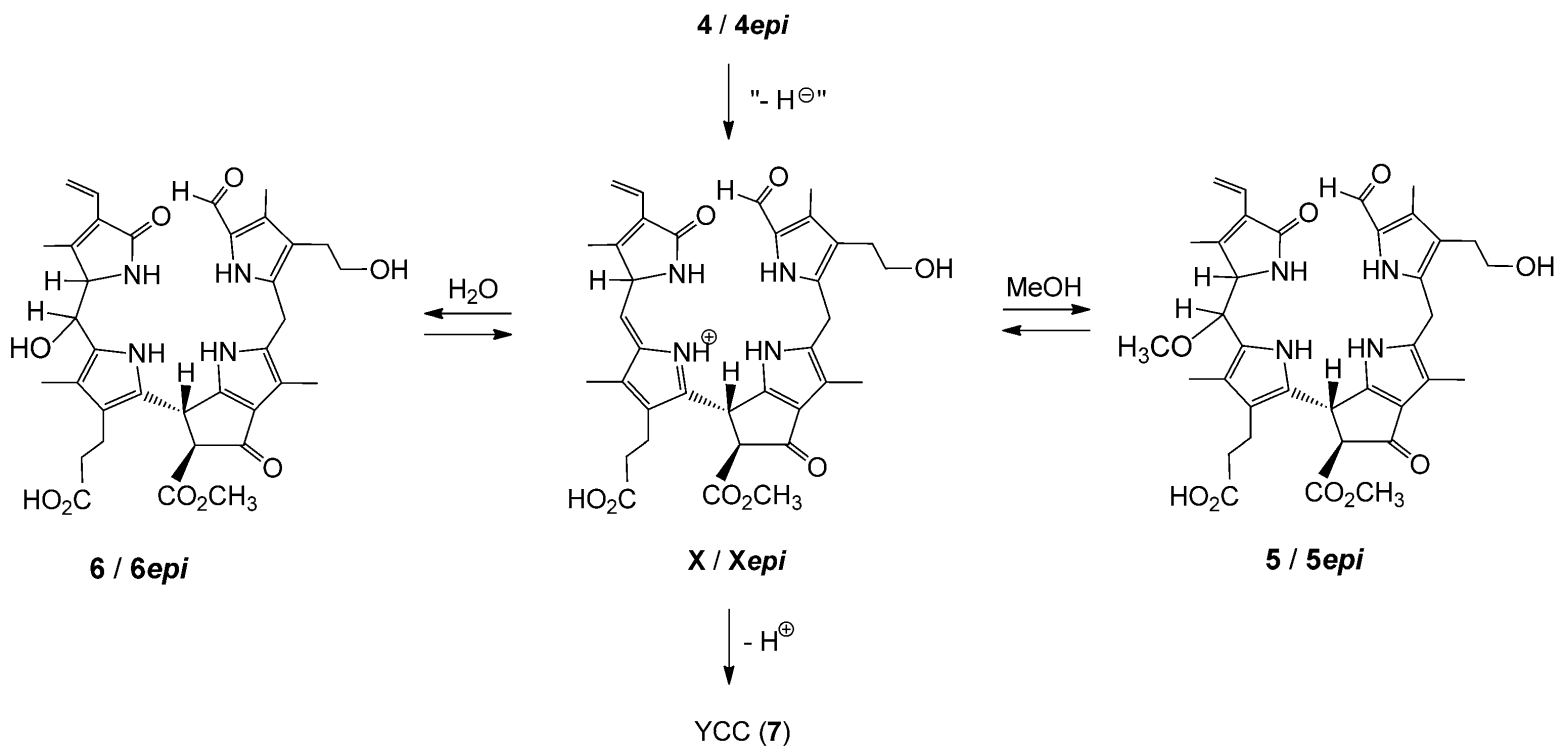
Possible oxidation mechanism: Oxidized NCCs 5/6 or 5*epi*/6*epi*, as well as YCC, may arise from the NCCs 4 or 4*epi* via the reactive azafulvalene-type intermediate X or X*epi*.

The derived structures and the identity of the oxidized NCCs (**5*epi*** and **6*epi***) from the “chemical” and the “biological” oxidation experiments of the NCC **4*epi***, indicated the same remarkable stereo- and regiochemical outcome of both highly selective paths. Formation of **5*epi*** and **6*epi*** by oxidation of **4*epi*** by DDQ would best be rationalized by occurring via the reactive aza-fulvalene-type intermediate **X*epi*** that is trapped by addition of a weak nucleophile, if available, such as methanol or water (see Scheme 5).

**Scheme 6:**
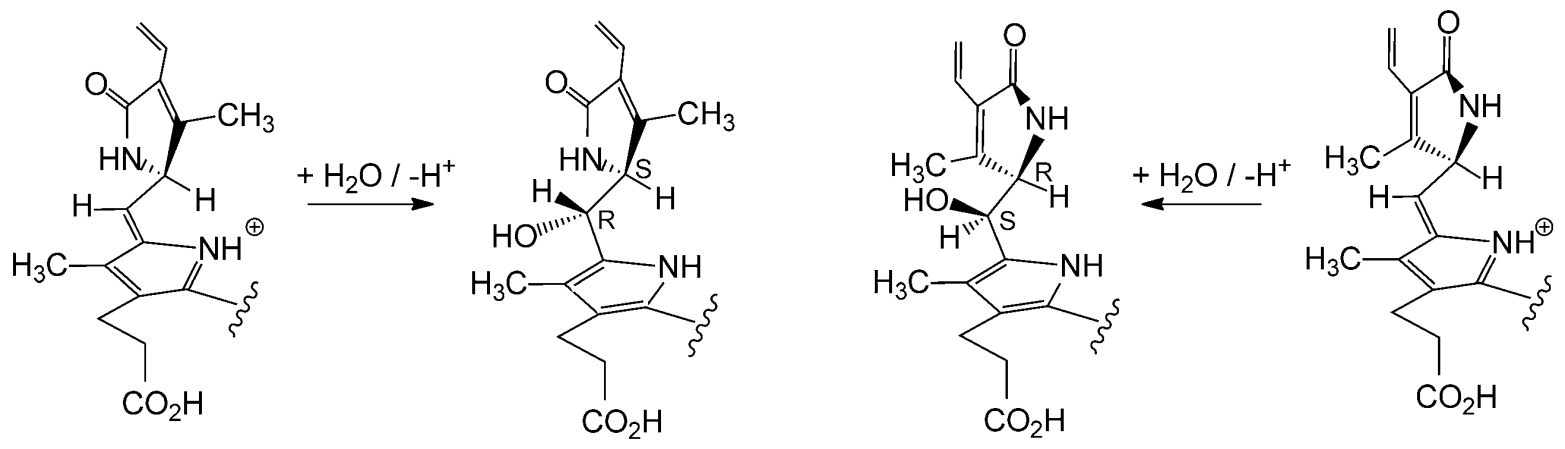
NCC oxidation: The configuration at C16 (of ring D) appears to determine the configuration in the addition of solvent molecules to C15 (the “*meso*-position”) under kinetically controlled conditions. NCCs 4 and 4*epi*, the precursors of X an X*epi*, resp., only differ by the absolute configuration at C16 (which has not yet been assigned).

Likewise, the very selective oxidation of **4** to **5** and **6** in the *Sp. wallisii* leaf homogenates would involve the analogous intermediate **X**. The observed diastereoselectivity could be deduced to be due to the adjacent substituted pyrrolinone unit (ring D), which may hinder the addition reaction at C15 to the face that is shielded by the H_3_C17^1^ group (see Scheme 6). As “chemical” and “biological” oxidations of **4** or **4*epi*** showed the same stereoselectivity, they may indicate the relevance of the same reactive (aza-fulvalene-type) intermediate on both oxidation paths, and thus, the noteworthy absence of enzyme-based contribution to the remarkable stereoselectivity in the final step of the formation of the oxidized NCCs **5** and **6**, or **5*epi*** and **6*epi***.

### v) Conversion of the 15-OH-*epi*NCC (6*epi*) to two stereoisomeric 15-OMe-*epi*NCCs in acidic methanolic solvent mixtures

**Figure 4:**
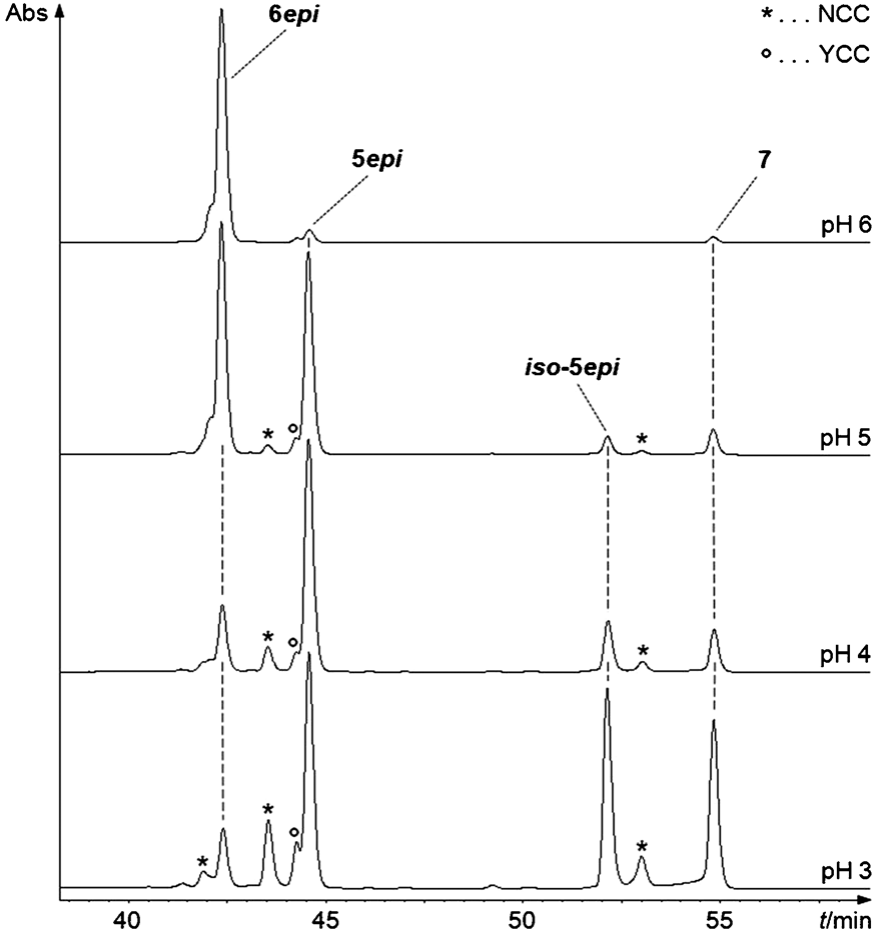
Conversion of 15-OH-*epi*NCC (6*epi*) to epimeric 15-OMe-*epi*NCCs (5*epi* and *iso*-5*epi*) and to YCC 7: HPLC analyses of acidic solutions of 6*epi* in 100 mm aq. buffer/MeOH 7/3 (v/v) that were stored for 5 h in the dark at ambient temperature. At pH 3, 6*epi* was largely consumed, giving similar amounts of 5*epi* and *iso*-5*epi* and of YCC 7.

Dilute acidic solutions of **6*epi*** in phosphate buffer/MeOH (7:3 (v/v)) were stored at room temperature for 5 h. Analysis of aliquots by HPLC showed pH-dependent formation of less polar NCCs, as well as of a YCC (see Figure 4). At pH 6 only about 5 % of **6*epi*** were selectively converted within 5 h to the less polar **5*epi***, at pH 5 roughly 50 % conversion to **5*epi*** had occurred, and at pH 4 about 90 % of **6*epi*** were converted, mostly to **5*epi***, and small amounts of an additional, still less polar NCC (named ***iso*****-5*epi***) were also formed, besides some YCC **7**. In a further 5 h experiment at pH 3, the less polar NCC ***iso*****-5*epi*** became nearly equally abundant as **5*epi***, and YCC **7** also accumulated to a larger extent.4

The more rapidly formed and more polar “new” NCC was identified as **5*epi***, as it had the same mass spectrometric and HPL-chromatographic characteristics as **5*epi*** (15-OMe-*epi*NCC) from “direct” oxidation of **4*epi*** with either *Sp. wallisii* leaf material, or with DDQ. At pH 5 **5*epi*** formed about 10 times faster than its presumed C15-epimer (named ***iso*****-5*epi***, see below), pointing to a sizable stereoselectivity in the indicated acid catalyzed substitution at C15 of a hydroxyl group by a methoxy group. This substitution process presumably proceeds via the intermediary aza-fulvalenium ion **X*epi*** (see Scheme 5), inferred above, strengthening the mechanistic interpretation of the stereoselectivity in the “chemical” and “biological” paths of the formation of **5*epi*** and **6*epi*** from **4*epi***.

An ESI-MS spectrum of ***iso*****-5*epi*** exhibited a pseudo-molecular ion at *m*/*z* 675.1, consistent with a molecular formula of C_36_H_42_N_4_O_9_, and confirming it as isomer of **5*epi*** (see Supporting Information, Figure S8). Fragments at *m*/*z* 643.2 and 611.3 also indicated loss of one and of two units of methanol, and effective loss of ring D (C_7_H_9_NO) was suggested again by a strong fragment at *m*/*z* 552.2. The ^1^H NMR spectrum of ***iso*****-5*epi*** in CD_3_OD at 25 °C (see Supporting Information, Figure S9) showed the same set of the characteristic signals, as the spectrum of **5*epi***, including a singlet at *δ*=3.11 ppm, assigned to the H_3_CO group at C15. Thus, C15 carries a methoxy substituent in ***iso*****-5*epi***, as in **5*epi***. The closely spaced signals, at 4.19 and 4.17 ppm, of HC15 and of HC16 generate an AB system, which shows NOE correlations with the singlet at 2.11 ppm of H_3_C17^1^. Further detailed information from multidimensional NMR (^1^H,^1^H-COSY and ROESY spectra, see Figure S9) suggest this new *epi*NCC to be a 1-formyl-3^2^-hydroxy-15-methoxy-19-oxo-16*epi*-16,19-dihydro-phyllobilane, that is, the 15-epimer of **5*epi*** (or ***iso*****-5*epi***) This NMR-derived structure indicates ***iso*****-5*epi*** to be related to **5*epi*** by exchange of the methoxy-group at C15 with inversion of configuration at this “meso′-carbon.

An additional fraction with lower polarity accumulated upon prolonged storage of the reaction mixture at low pH, which was identified (UV/Vis and mass spectra, HPLC) as the yellow Chl catabolite (YCC) **7**, the apparent product of acid induced elimination of methanol from ***iso*****-5*epi*** and from **5*epi***.

Selective formation of **5** and **6** from **4**, as well as of **5*epi*** and **6*epi*** from **4*epi*** indicates retention, in these oxidation reactions, of the H atom at C16, also retaining the configuration at C16. Furthermore, the observed similar ^1^H- and ^13^C NMR chemical shift values of the oxidized NCCs **5**, **5*epi*** and **6*epi*** (see Table 1), would be consistent with a common relative configuration at C15 and C16. This is also inferred, based on the pathway involving the electrophilic azafulvalene intermediate **X/Xepi** (see Scheme 5). Accordingly, in the absence, so far, of an assignment of the absolute configuration of C16 in the NCCs **4** and **4*epi***, the oxidized NCCs of **5** and **6**, resp. **5*epi*** and **6*epi***, would either be 15*R*,16*S* or 15*S*,16*R*. On the other hand, the exceptional shift to 2.11 ppm of the methyl group singlet of H_3_C17^1^ in the spectrum of ***iso*****-5*epi***, would now be consistent with a different relative configuration at the two chiral carbons C15 and C16, presumably due to an inverted configuration at C15.

**Table 1:** ^1^H NMR chemical shift values in CD_3_OD of 15-OMe-NCC 5 (600 MHz, 10 °C) and the two 15-OMe-*epi*NCCs 5*epi* and *iso*-5*epi* (both 500 MHz, 25 °C). Significant chemical shift differences are found in the vicinity of C15 and are highlighted as bold numbers

	5	5*epi*	*iso*-5*epi*
C2^1^	2.20	2.29	2.24
C3^1^	2.59	2.65	2.62
C3^2^	3.46	3.58	3.48
C5	3.92	3.96	3.96
C7^1^	2.16	2.14	2.08
C8^5^	3.76	3.77	3.75
C10	4.96	4.97	4.92
C12^1^	2.65/2.79	2.68/2.78	2.65/2.73
C12^2^	2.34	2.36	2.26/2.31
C13^1^	1.95	2.01	1.97
C15	**3.78**	**3.88**	**4.19**
C15^2^	3.12	3.07	3.11
C16	**4.10**	**4.28**	**4.17**
C17^1^	**1.06**	**1.42**	**2.11**
C18^1^	6.37	6.39	6.43
C18^2^	5.35/6.12	5.35/6.12	5.34/6.06
C20	9.28	9.40	9.32

### vi) Acid induced formation of the yellow Chl-catabolite 7 by elimination of water from 15-OH-NCC 6*epi*

**Figure 5:**
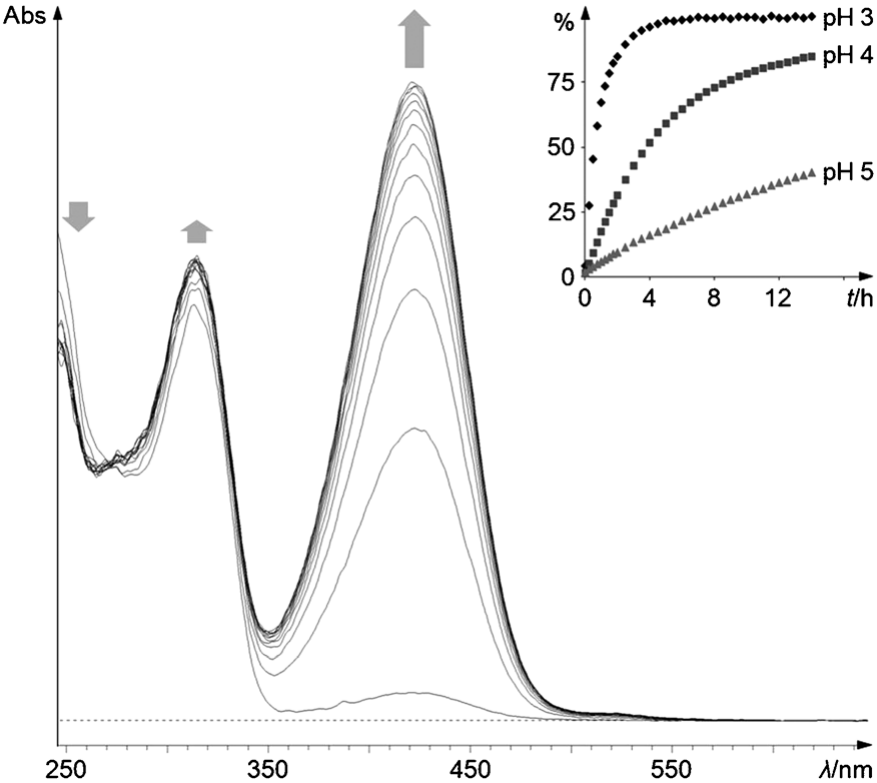
Kinetic analysis of the acid induced conversion of 15-hydroxy-*epi*NCC (6*epi*) to YCC 7. A solution of 6*epi* in 100 mm aq. potassium phosphate (pH 3.0)/ACN 7:3 (v/v) was stored at room temperature in the dark. Formation of YCC 7 in the pH 3 experiment was monitored by UV/Vis spectra, recorded at the start and then every 30 min. The arrows indicate the direction of the change of the absorption of the acidic solution in the first 6 h. Inset: Kinetic analysis of the transformation of 6*epi* to YCC 7 in experiments at pH 3, 4 and 5.

Upon storage in weakly acidic medium, **6*epi*** underwent water elimination readily, and converted cleanly into YCC **7** (see Figure 5). To avoid formation of a 15-methoxy-NCC (such as **5*epi***, see above), mixtures of aqueous buffers and acetonitrile 7:3 (v/v) were used. The increase of the absorbance at approximately 425 nm monitored by periodically recorded UV/Vis spectroscopy was used to quantify formation of the YCC **7**. Storage of the solution of **6*epi*** showed 50 % conversions to **7** within about 35 min at pH 3, about 227 min at pH 4, and about 20 h at pH 5. Loss of water from C15, which is directly bound to the α-position of a pyrrole unit, is acid induced and exhibits a sizeable effective rate increase (of roughly 5 times) per unit of decreasing pH value. The observed ease of the elimination of water in slightly acidic aqueous medium can be rationalized by the formation of the stabilized aza-fulvalenium-type intermediate **X*epi*** (see Scheme 4). This chemical finding would suggest this path of the formation of YCCs from oxidized NCCs (such as **4** and **4*epi***) to also occur with significant rates in (the acidic vacuoles of) de-greened leaves.

### vii) Exploratory studies of prerequisites for selective oxidation of NCCs 4 and 4*epi*

In order to gain further insights into the unprecedented, hypothetical oxidative process, conditions of incubation and of extract-preparation of senescent *Sp. wallisii* leaves were studied by HPLC-analysis of the reaction mixtures and on-line UV/Vis detection.

#### Effect of temperature

When the *Sp. wallisii* extract was prepared at ambient temperature and in the presence of air, HPLC analysis showed a dominant FCC (**2**) and several minor NCC fractions (mostly **4**, **5** and **6**). In contrast, when an extract of senescent *Sp. wallisii* leaves was prepared from leaves frozen with liquid nitrogen, formation of the NCCs **5** and **6** was insignificant (even in the presence of air, see Figure 1). As expected, the temperature of the ground leaf mixture was a critical factor, and oxidation of **4** to **5** and **6** was severely retarded at low temperatures.

#### Composition of the gaseous sphere

**Figure 6:**
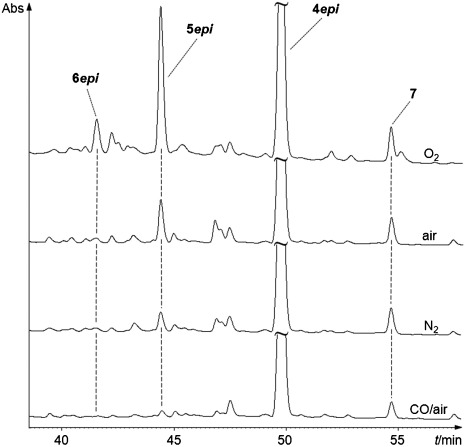
HPL-chromatograms of analytical experiments with *Spathiphyllum wallisii* leaf material showing the oxidation of artificially added NCC 4*epi* to oxidized NCCs 5*epi* and 6*epi*, and YCC 7, under different experimental conditions. Solutions containing the NCC 4*epi* were first purged with specified gases (O_2_, air, N_2_, CO/air 1/1 (v/v)), and then mixed with deoxygenated *Sp. wallisii* leaf material. After storage under the indicated conditions for half an hour in the dark at ambient temperature the slurries were centrifuged and supernatants analyzed by HPLC. Formation of oxidized NCCs (6*epi* and 5*epi*), as well as of YCC 7, was monitored by UV/Vis detection at 320 nm. Experiments are shown (top to bottom) from purging with O_2_, with air, with N_2_, and with CO/air 1:1 (v/v), and which indicate decreasing activity, in this order.

**O_2_**: A ground sample of a green *Sp. wallisii* leaf was first evacuated and a solution of **4*epi*** in buffer (50 mm, pH 7)/methanol (3:7 v/v), purged with oxygen gas, was added. The reaction flask was purged with O_2_ and the mixture was stored for 30 min in the dark at room temperature. HPLC analysis of the supernatant showed significantly higher amounts of **5*epi*** and **6*epi*** related to the control experiment, in which the reaction mixture was prepared and stored under air (see Figure 6). In an experiment that was carried out similarly, but with a deoxygenated solution of the NCC **4*epi*** and keeping the reaction mixture under inert gas, the conversion of **4*epi*** to **5*epi*** and **6*epi*** was strongly diminished. These findings indicated the presence of oxygen in the ground leaf mixture to be critical, and suggested oxidation of **4** to the NCCs **5** and **6** to be severely retarded under inert atmosphere.6

**CO**: In a similar experiment, an evacuated, ground sample of a green *Sp. wallisii* leaf was first treated with a de-aerated solution of the NCC **4*epi***, and the reaction mixture was then purged with carbon monoxide/air 1:1 (v/v). The conversion of **4*epi*** to **5*epi*** and **6*epi*** was strongly diminished (see Figure 6). This finding suggested the presence of carbon monoxide in the mixture to inhibit oxidation of **4*epi*** to the NCCs **5*epi*** and **6*epi***.

#### Effect of cyanide or hydroxide ions

Likewise, addition of ca. 3 mm aqueous solutions of potassium cyanide, or of potassium hydroxide, to the freshly ground plant material at room temperature and under air, indicated strongly diminished formation of **5** and **6** from the endogenous NCC **4** (see Supporting Information, Figure S10).

#### Effects of solvents

**Absence of MeOH**: Soaking of ground lyophilized leaf material for five minutes in water (at ambient temperature in the dark) led to formation of **6** (whereas **5** was hardly detected). The same result (formation of **6**, but not of **5**) was observed, when ground (water containing) fresh leaf sections were stored in the dark for five minutes, before HPLC analysis. Likewise, when in the extraction acetone was used instead of methanol, **6** again was found (but **5** was not detected by HPLC analysis). Methanolic extraction of lyophilized leaves gave little detectable formation of C15 derivatives of **4** (see Supporting Information, Figure S11).

**The elusive “oxidative activity” requires soluble and insoluble components for activity**: A piece of a senescent *Sp. wallisii* leaf was ground in a mortar at ambient temperature. Then the resulting slurry was centrifuged and the supernatant was separated from the remaining pellet. NCC **4*epi*** was added under air to both samples, which were left to stand at room temperature for 30 min. HPLC-analyses showed strongly diminished formation of the NCC derivatives (see Supporting Information, Figure S12). When ground leaf pieces were heated to 70 °C for 10 min before addition of the (exogenous) NCC **4*epi***, oxidation of **4*epi*** to **5*epi*** and **6*epi*** was insignificant in the presence of air (see Supporting Information, Figure S12). These experiments suggest the requirement for components contained in both, the pellet and the supernatant, and that the elusive “oxidative activity” was inactivated by the short heat treatment.

## Conclusions and Outlook

In extracts of a variety of yellow, senescent leaves, colorless NCCs and DNCCs were found to accumulate, and appeared to represent the “final” stages of metabolically controlled Chl breakdown. Typically, the content of these Chl catabolites was observed to decrease at later stages of senescence, and traces of yellow and pink Chl catabolites (YCCs[13,14] and PiCCs,[13] respectively, see Scheme 2) were found in several studies. So far, these observations have not found any rational explanation. However, as reported here, NCCs may be oxidized by an endogenous oxidative pathway, which provides functionalized NCCs that are prone to undergo elimination to YCCs. YCCs, in turn, are readily oxidized to PiCCs, a process that occurs spontaneously in the presence of air, and which is assisted by biologically relevant metal ions, such as Zn^II^ ions.[17] PiCCs appear to undergo various further, still uncharacterized reactions - a preliminary observation that is under investigation in our labs.

The here described oxidative path involving NCCs may open a natural route to further breakdown via the colored Chl catabites, and to eventual degradation, altogether, of tetrapyrrolic phyllobilins. Similar oxidative transformations of DNCCs, the dioxobilin analogues of NCCs,[6, 18] may provide DYCCs and their further descendants. Recent studies in our labs have, indeed, provided evidence for the occurrence of DYCCs in extracts of yellow senescent grapevine leaves.[14d]

Our preliminary “biochemical” experiments indicate the oxidation of NCCs in leaf homogenates to be associated with heat de-naturable components of the leaf material, and to be inhibited by CO, suggesting the relevance of (metallo)enzyme catalyzed processes. Clearly, it remains to examine this aspect further and to also provide evidence for the occurrence of such transformations in intact (senescent) leaves, as well as for their cellular location. Interestingly, homogenates of green *Sp. wallisii* leaves also oxidize NCCs, indicating the “oxidative activity” to play a role independent of the senescence syndrome. Our studies also call for caution with respect to storage and work-up of senescent leaves, in particular, if a quantitative interpretation of the presence of the Chl-catabolites is a goal of the study.

Oxidative transformations are considered here as part of the “late stages of the breakdown” of Chl. These would thus contribute to the loss of colorless phyllobilanes in senescent leaves, and produce linear tetrapyrroles with conjugated systems extended over two or more of the pyrrolic rings. Such “oxidized” phyllobilanes (as well as the persistent *hm*FCCs[10]) feature structures and chromophores that may also enable them to function as new molecular tools for “non-invasive studies” of senescence processes in leaves and fruit.[17] Furthermore, the chromophores of such “oxidized” phyllobilanes resemble those of some bilins, pigments from heme breakdown with important biological functions,[19] thus calling for attention as to the still elusive biological functions of phyllobilins,[3b] the growing class of bilin-type tetrapyrroles derived from chlorophyll.

## Experimental Section

### Materials

*Plant material:* Yellow senescent and fresh green leaves of the Peace Lily (*Spathiphyllum wallisii*) were both harvested from the same home-grown plant in Innsbruck, Austria. They were transported at ambient temperature to the University, where they were frozen and stored at −80 °C, until they were analyzed.

*Chemicals:* HPLC grade methanol (MeOH) and acetic acid 100 % were from VWR (West Chester, USA), HPLC grade *n*-hexane from *Arcos Organics* (Geel, Belgium), HPLC grade acetonitrile (ACN) from Sigma–Aldrich (St. Louis, USA), acetone puriss. p.a. from Fluka (Buchs, CH), ultrapure water (18 MΩ.cm^−1^) from a *Millipore* apparatus. Dichloromethane (CH_2_Cl_2_)≥99 %, puriss., from Sigma–Aldrich (St. Louis, USA), was freshly distilled and filtered over Alox before use. Phosphoric acid 85 %, purum p.a., potassium phosphate monobasic, puriss. p.a., potassium phosphate dibasic, puriss. p.a., ammonium acetate, puriss. p.a., and 2,3-dichloro-5,6-dicyano-1,4-benzoquinone (DDQ) were from *Fluka* (Buchs, CH). 1 g and 5 g SepPak C18 cartridges were from Waters Associates (Milford, USA).

### Methods

*HPLC: Analytical HPLC:* Shimadzu HPLC system, with manual sampler, DGU-20A5 online degasser, LC-20AD pump, CBM-20A system controller, SPD-M20A diode array detector, Jasco FP-920 fluorescence detector, a Rheodyne injection valve with 20 or 200 μL loop. Data were collected and processed with Shimadzu LC Solution. Phenomenex Hyperclone ODS 5 μm 250×4.6 mm i.d. column connected to a Phenomenex ODS 4×3 mm i.d. pre-colomn used at room temperature with a flow rate of 0.5 mL min^−1^. Solvent system: solvent A: 50 mm aq. potassium phosphate (pH 7.0), solvent B: MeOH; solvent composition (A/B) as function of time: analytical and co-injection experiments: 0–32 min: 55:45 to 39:61; 32–37 min: 39:61 to 0:100; 37–45 min: 0:100; 45–50 min: 0:100 to 55:45; analytical experiments with **4*epi***: 0–22 min: 55:45 to 44:56; 22–25 min: 44:56 to 0:100; 25–30 min: 0:100; 30–35 min: 0:100 to 55:45 or: 0–5 min: 80:20; 5–55 min: 80:20 to 30:70; 55–60 min: 30:70 to 0:100; 60–70 min: 0.100; 70–75 min: 0:100 to 80:20.

*Preparative HPLC:* Dionex Summit HPLC system, with manual sampler and online degasser, P680 pump, UVD340U diode array detector and a Rheodyne injection valve with 1 mL loop. Data were collected and processed with Chromeleon V6.50. Phenomenex Hyperclone ODS 5 μm 250×21.2 mm i.d. colomn connected to a ODS pre-column used at room temperature with a flow rate of 5 mL min^−1^. Solvent system: solvent A: 50 mm aq. potassium phosphate (pH 7.0), solvent B: MeOH; solvent composition (A/B) as function of time: isolation of **5**: 0–2 min: 65:35; 2–90 min: 65:35 to 40:60; 90–100 min: 40:60 to 0:100; 100–110 min: 0:100; 110–120 min: 0:100 to 65:35; isolation of **6*epi***: 0–5 min: 70:30; 5–90 min: 70:30 to 40:60; 90–100 min: 40:60 to 0:100; 100–110 min: 0:100; 110–120 min: 0:100 to 70:30.

*MPLC:* Büchi MPLC system equipped with two C-605 pumps, a C-615 pump manager and a C-630 UV-detector. A 450×35 mm i.d. colomn packed with EUROPREP 60–30 C18 60 Å 20–45 μm irregular was used at room temperature with a flow rate of 10 mL min^−1^. Solvent system: 50 or 100 mm aq. potassium phosphate (pH 7.0)/MeOH.

*LC/ESI-MS:* LC Packings Ultimate HPLC system, with manual sampler and helium degasser. UVD340U diode array detector and a Rheodyne injection valve with 30 μL loop. Data were collected and processed with Chromeleon V6.50. Phenomenex Hyperclone ODS 5 μm 250×4.6 mm i.d. colomn connected to a Phenomenex ODS 4×3 mm i.d. pre-colomn used at room temperature with a flow rate of 0.5 mL min^−1^. Solvent system: solvent A: 10 mm aq. ammonium acetate, solvent B: MeOH; solvent composition (A/B) as function of time: 0–85 min: 65:35 to 32:68; 85–88 min: 32:68 to 0:100; 88–96 min: 0:100; 96–100 min: 0:100 to 65:35. Coupled with Finnigan MAT 95*-*S or Finnigan LCQ Classic mass spectrometer (conditions see below).

*pH values:* Measured by using a WTW Sentix 21 electrode connected to a WTW pH 535 digital pH meter.

### Spectroscopy

*UV/Vis: λ*_max_ [nm] (log*ε* or *ε*_rel_), Hitachi U-3000 spectrophotometer, solvents: MeOH or (online) HPLC elution mixtures; concentrations of NCCs were calculated using the published extinction coefficients of **4*epi*** at 312 nm (log*ε*=4.23[15, 19]) and of YCC **7** (*Cj*-YCC) at 426 nm (log*ε*=4.51[12a]).

CD: λ_min/max_ [nm] (Δ*ε*), Jasco J715, solvent: MeOH.

NMR*: δ* [ppm], *J* [Hz], Bruker UltraShield Avance II+ 600 MHz or Varian Unity Inova 500 MHz; ^1^H,^1^H-homonuclear (COSY, ROESY) and ^1^H,^13^C-heteronuclear (HSQC, HMBC) experiments;[20] 10 °C or 25 °C; residual solvent peaks (CD_2_HOD: *δ*_H_=3.31 ppm, *δ*_C_=49.00 ppm; CD_2_HCN: *δ*_H_=1.94 ppm, *δ*_C_=1.32 ppm)[21] were used as internal references; signals are classified as singlet (s), doublet (d), double doublet (dd), triplet (t) and multiplet (m); broad=br.

ESI-MS*:*[22] *m*/*z* (rel. abundance; type of ion); signals due to isotopomers and their relative intensities are shown for base peaks and [*M*+H]^+^ pseudo molecular ions; Finnigan MAT 95-S or Finnigan LCQ Classic mass spectrometer, ESI source; positive ion mode, spray voltage 1.4 (Finnigan MAT 95) or 4.5 kV (Finnigan LCQ Classic).

**Isolation and characterization of non-fluorescent chlorophyll catabolites from senscent**­ ***Spathiphyllum wallisii***­ **leaves**: 5 g (wet weight) of yellow-greenish senescent leaves of the Peace Lily (*Spathiphyllum wallisii*) were mixed with about 1 g of sea sand and ground to a fine slurry at ambient temperature. After addition of 10 mL of MeOH and further grinding, the liquid supernatant was decanted and the extraction was repeated three times with 5 mL of MeOH each. The collected methanolic extracts were centrifuged (5 min, 5000 rpm) and the resulting yellow-brownish solution (about 20 mL) was extracted two times with n-hexane. After dilution with 130 mL of 50 mm aq. potassium phosphate (pH 7.0) the clear solution was applied to a pre-conditioned 5 g SepPak cartridge and rinsed with 30 mL of H_2_O. The NCC-containing fraction was eluted with 5 mL of MeOH, dried under reduced pressure and dissolved in 500 μL of MeOH. The dark brown solution was diluted 1:1 (v/v) with 50 mm aq. potassium phosphate (pH 7.0) and applied to preparative HPLC after centrifugation (5 min, 13 000 rpm). The NCC or YCC containing fractions were collected. The dominant fraction with a retention time of 26 min was reduced to 10 mL using a rotary evaporator, diluted 1:1 (v/v) with 50 mm aq. potassium phosphate (pH 7.0) and applied to a pre-conditioned 1 g SepPak cartridge. After rinsing with 20 mL of H_2_O a slightly yellow fraction was eluted with 5 mL of MeOH. The solvent was evaporated in vacuo to give about 250 μg (370 nmol) of **5**. The dried sample was used for spectroscopic analyses and all further experiments. Fractions of minor catabolites (**6**, **4** and **7**, see below) were characterized by their UV/Vis absorption, mass spectrometric fragmentation patterns and their HPLC elution properties.

#### Spectroanalytical data (for numbering see Figure 7)

*1-Formyl-3^2^-hydroxy-15-methoxy-19-oxo-16n-16,19-dihydrophyllobilane* (***5**):* UV/Vis (*c*=3.4×10^−5^ m): *λ*_max_ (*ε*_rel_)=312 (1.00), 239 nm (sh, 1.49); CD (*c*=3.4×10^−5^ m): *λ*=313 (3.0), 279 (−18.2), 255 (−8.3), 252 (−8.4), 228 nm (11.7); ^1^H NMR (600 MHz, 10 °C, CD_3_OD): *δ*=1.06 (s, H_3_C17^1^), 1.95 (s, H_3_C13^1^), 2.16 (s, H_3_C7^1^), 2.20 (s, H_3_C2^1^), 2.34 (m, H_2_C12^2^), 2.59 (br t, *J*∼7.0, H_2_C3^1^), 2.65 (m, H_A_C12^1^), 2.79 (m, H_B_C12^1^), 3.12 (s, H_3_C15^2^), 3.46 (m, H_2_C3^2^), 3.76 (s, H_3_C8^5^), 3.78/4.10 (AB-system, *J*=9.2, HC15 and HC16), 3.92 (br s, H_2_C5), 4.96 (s, HC10), 5.35 (dd, *J*=2.1/11.7, H_A_C18^2^), 6.12 (dd, *J*=2.2/17.8, H_B_C18^2^), 6.37 (dd, *J*=11.7/17.8, HC18^1^), 9.28 ppm (s, HC20); LC/ESI-MS: *m*/*z* (%): 751.3 (2) [*M*−H+2K]^+^, 713.3 (40) [*M*+K]^+^, 697.3 (2) [*M*+Na]^+^, 677.3 (1), 676.4 (2), 675.4 (4) C_36_H_43_N_4_O_9_^+^, [*M*+H]^+^, 643.3 (10) [*M*−CH_3_OH+H]^+^, 611.2 (2) [*M*−2CH_3_OH+H]^+^, 554.2 (8), 553.2 (30), 552.2 (100), [*M*−ring D+H]^+^, 520.2 (3) [*M*−ring D−CH_3_OH+H]^+^ (see Figure 2).7

**Figure 7:**
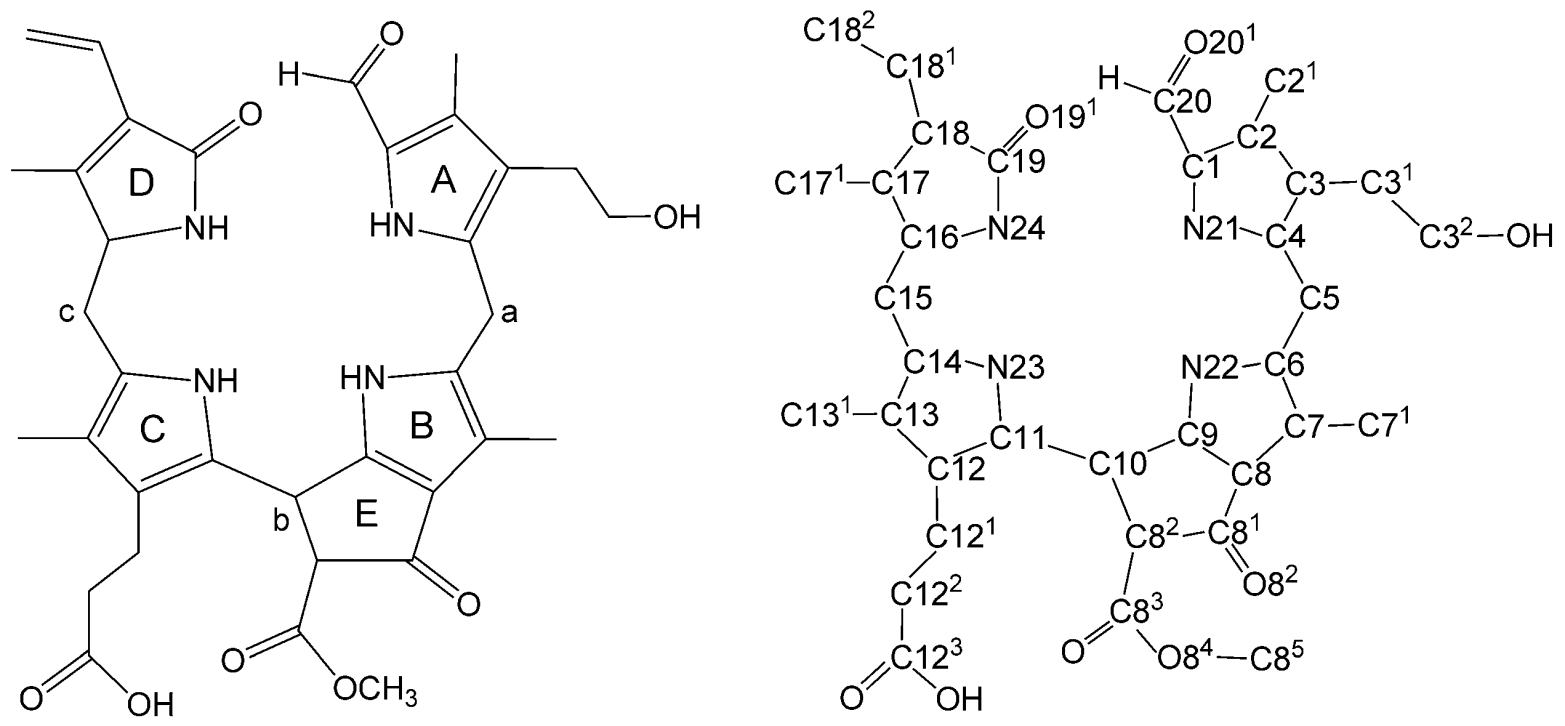
Labels of rings (left) and atom numbering (right) of phyllobilanes, exemplified for the NCC 4, a 1-formyl-19-oxo-3^2^-hydroxy-16,19-dihydro-phyllobilane.

*1-Formyl-3^2^,15-dihydroxy-19-oxo-16n-16,19-dihydrophyllobilane (**6***): UV/Vis (online, 50 mm aq. potassium phosphate (pH 7.0)/MeOH about 45:55 (v/v)): *λ*_max_ (*ε*_rel_)=314 (1.00), 245 nm (sh, 1.36); LC/ESI-MS: *m*/*z* (%): 699.3 (56) [*M*+K]^+^, 663.3 (3), 662.3 (8), 661.3 (18) C_35_H_41_N_4_O_9_^+^, [*M*+H]^+^, 643.3 (20), [*M*−H_2_O+H]^+^, 611.3 (6) [*M*−CH_3_OH-H_2_O+H]^+^, 540.2 (22), 539.2 (37), 538.2 (100) [*M*−ring D+H]^+^, 506.2 (18) [*M*−ring D−CH_3_OH+H]^+^ (see Figure 2).

*1-Formyl-3^2^-hydroxy-19-oxo-16,19-dihydrophyllobilene-c (YCC **7***) from senescent *Sp. wallisii* leaves: UV/Vis (online, 50 mm aq. potassium phosphate (pH 7.0)/MeOH about 35:65 (v/v)): *λ*_max_ (*ε*_rel_)=426 (1.05), 313 (1.00), 244 nm (sh, 1.16); LC/ESI-MS: *m*/*z* (%): 681.2 (20) [*M*+K]^+^, 645.2 (9), 644.2 (35), 643.2 (100) [C_35_H_38_N_4_O_8_]^+^ [*M*+H]^+^, 611.2 (22) [*M*−CH_3_OH+H]^+^.

**HPLC analysis of partial oxidation of 4*epi*, when added to complete**­ ***Sp. wallisii***­ **leaf extracts**: A yellow-greenish senescent leaf part (3 cm^2^, about 200 mg wet weight, *Spathiphyllum wallisii*) was ground with a small amount of sea sand in a mortar at ambient temperature. The plant material was transferred into a small reaction tube and 700 μL of a 5×10^−5^ m solution of **4*epi*** in MeOH/50 mm aq. potassium phosphate (pH 7.0) 7:3 (v/v) were added. The slurry was stored in the dark at ambient temperature and was shaken from time to time. After 30 min the mixture was centrifuged (3 min, 13 000 rpm) and the supernatant was diluted 1:1 (v/v) with 50 mm aq. potassium phosphate (pH 7.0). After a second round of centrifugation (5 min, 13 000 rpm) the resulting yellow extract was used for analysis by HPLC (using detection at 320 nm). Polar NCCs, **5*epi*** and **6*epi***, and **4*epi*** were the major fractions, and minor amounts of the isomeric NCCs **4 n**, **5 n** and **6 n** were also detected under these conditions (see Figure 3). In a similar experiment with an extract of a green *Sp. wallisii* leaf (see below for preparative oxidation of **4*epi*** with plant material) oxidation of **4*epi*** was observed (qualitatively) to the same products, as seen when senescent leaves were used (see Figure 6).

**Semi-preparative oxidation of 4*epi* in the extract of a**­ ***Sp. wallisii***­ **leaf**: A piece of a green *Spathiphyllum wallisii* leaf (25 cm^2^) and a small amount of sea sand were ground in a mortar at ambient temperature, and the resulting slurry was transferred into a 10 mL reaction flask using two 1 mL portions of water. A solid sample of **4*epi*** (12.5 mg, 19.4 μmol) was mixed with 3 mL of water (using an ultrasonic bath) and the slightly yellow suspension was added to the slurry of the ground leaf. The mixture was stirred at ambient temperature in the dark for 6 h. Then 15 mL of MeOH were added and the mixture was filtered through a Büchner funnel. The remaining residue was extracted twice with 2 mL of MeOH, each. The collected green fractions were extracted with two portions of n-hexane (10 mL each), and the resulting brown methanolic solution was concentrated to 500 μL at reduced pressure using a rotary evaporator. After addition of 500 μL of MeOH and centrifugation for 5 min at 13 000 rpm, the dark brown solution was removed and used for preparative HPLC. Four NCC- and one YCC-containing fractions (from UV/Vis) were collected, quantified via UV/Vis spectrometry, and identified by mass spectrometric and HPLC analysis. About 2.7 μmol of **4*epi*** could be re-isolated in this way, as well as about 10.7 μmol (55 %) of the more polar NCCs **6*epi*** and **5*epi***, and (smaller amounts of) YCC **7**.

The fraction with a retention time of 50 min in the preparative HPLC experiment (containing **6*epi***) was reduced to 10 mL using a rotary evaporator, diluted 1:1 (v/v) with 50 mm aq. potassium phosphate (pH 7.0) and applied to a pre-conditioned 1 g SepPak cartridge. After rinsing with 20 mL of H_2_O a slightly yellow fraction was eluted with 5 mL of MeOH. The solvent was reduced to 500 μL and after 1:1 (v/v) dilution with MeOH the fraction was applied to the preparative HPLC system in the same way as described above once more. The **6*epi*** containing fraction was collected, reduced to half of its volume using a rotary evaporator, diluted 1:1 (v/v) with 50 mm aq. potassium phosphate (pH 7.0) and applied to a pre-conditioned 1 g SepPak cartridge. After rinsing with 20 mL of H_2_O the NCC containing fraction was eluted with 5 mL of MeOH. The solvent was evaporated in vacuo to give 1.9 mg (2.9 μmol) of **6*epi***. The dried sample was used for spectroscopic analyses and all further experiments. MeO-NCC **5*epi*** and minor other fractions were tentatively characterized by mass spectrometric analyses, their UV/Vis absorbance and their HPLC elution properties.

#### Spectroanalytical data

*1-Formyl-3^2^-hydroxy-15-methoxy-19-oxo-16epi-16,19-dihydrophyllobilane* (**5*epi***): UV/Vis (online, 50 mm aq. potassium phosphate (pH 7.0)/MeOH about 50:50 (v/v)): *λ*_max_ (*ε*_rel_)=314 (1.00), 241 nm (sh, 1.30); LC/ESI-MS: *m*/*z* (%): 751.1 (20) [*M*−H+2K]^+^, 735.2 (6) [*M*−H+K+Na]^+^ 713.3 (57) [*M*+K]^+^ 697.3 (16) [*M*+Na]^+^, 677.0 (6), 676.0 (23), 675.0 (53) C_36_H_43_N_4_O_9_^+^, [*M*+H]^+^, 645.3 (9), 644.2 (44), 643.3 (100) [*M*−CH_3_OH+H]^+^, 611.3 (20) [*M*−2CH_3_OH+H]^+^, 552.2 (67) [*M*−ring D+H]^+^, 520.2 (12) [*M*−ring D-CH_3_OH+H]^+^.

*1-Formyl-3^2^,15-dihydroxy-19-oxo-16epi-16,19-dihydrophyllobilane (**6epi***): UV/Vis (*c*=2.3×10^−5^ m): *λ*_max_ (*ε*_rel_)=314 (1.00), 242 nm (sh, 0.97); CD (*c*=2.3×10^−5^ m): *λ*=311 (3.0), 281 (−15.4), 260 (sh, −2.5), 252 (sh, 1.3), 229 nm (35.5); ^1^H NMR (600 MHz, 10 °C, CD_3_OD): *δ*=1.52 (s, H_3_C17^1^), 2.00 (s, H_3_C13^1^), 2.11 (s, H_3_C7^1^), 2.27 (s, H_3_C2^1^), 2.33 (t, *J*=7.0, H_2_C12^2^), 2.62 (br t, *J*∼6.9, H_2_C3^1^), 2.66 (m, H_A_C12^1^), 2.74 (m, H_B_C12^1^), 3.46 (m, H_2_C3^2^), 3.74 (s, H_3_C8^5^), 3.93 (br s, H_2_C5), 4.24/4.38 (AB system, *J*=7.5, HC16 and HC15), 4.92 (s, HC10), 5.34 (dd, *J*=2.0/11.7, H_A_C18^2^), 6.12 (dd, *J*=2.0/17.8, H_B_C18^2^), 6.40 (dd, *J*=11.6/17.7, HC18^1^), 9.38 ppm (s, HC20); ^13^C NMR (600 MHz, 10 °C, CD_3_OD): *δ*=8.6 (2^1^), 9.0 (7^1^), 9.1 (13^1^), 12.3 (17^1^), 21.6 (12^1^), 23.6 (5), 27.9 (3^1^), 37.0 (10), 39.1 (12^2^), 52.4 (8^5^), 62.3 (3^2^), 65.9 (16), 67.8 (8^2^), 69.2 (15), 112.0 (7), 115.8 (13), 119.0 (18^2^), 120.6 (3), 121.1 (12), 125.2 (11), 125.9 (8), 126.8 (18^1^), 127.9 (14), 129.3 (1), 129.9 (18), 134.4 (6), 134.9 (2), 138.4 (4), 154.6 (17), 160.9 (9), 171.5 (8^3^), 174.9 (19), 177.5 (20), 180.9 ppm (12^3^); LC/ESI-MS: *m*/*z* (%): 737.2 (13) [*M*−H+2K]^+^, 721.3 (5) [*M*−H+K+Na]^+^, 699.2 (20) [*M*+K]^+^, 683.2 (12) [*M*+Na]^+^, 663.1 (14), 662.0 (46), 661.1 (100) C_35_H_41_N_4_O_9_^+^, [*M*+H]^+^, 643.2 (16) [*M*−H_2_O+H]^+^, 611.1 (5) [*M*−CH_3_OH−H_2_O+H]^+^, 538.1 (35) [*M*−ring D+H]^+^, 506.2 (12) [*M*−ring D-CH_3_OH+H]^+^.

*1-Formyl-3^2^-hydroxy-19-oxo-16,19-dihydrophyllobilene-c* (**7**) from *Sp. wallisii* oxidation of the NCC **4*epi***: UV/Vis (online, 50 mm aq. potassium phosphate (pH 7.0)/MeOH about 35:65 (v/v)): *λ*_max_ (*ε*_rel_)=427 (1.46), 313 (1.00), 246 nm (0.76); LC/ESI-MS: *m*/*z* (%): 757.2 (5) [*M*−2H+3K]^+^, 719.2 (12) [*M*−H+2K]^+^, 703.1 (5) [*M*−H+K+Na]^+^, 681.3 (9) [*M*+K]^+^, 665.2 (5) [*M*+Na]^+^, 645.2 (10), 644.2 (42), 643.3 (100) C_35_H_39_N_4_O_8_^+^, [*M*+H]^+^, 611.2 (19) [*M*−CH_3_OH+H]^+^.

**Preparation of 5*epi* and 6*epi* via oxidation of 4*epi* with 2,3-dichloro-5,6-dicyano-1,4-benzoquinone (DDQ)**: A solution of 201.4 mg (312.4 μmol) of **4*epi*** in acetone at −79 °C was treated with 78 mg (1.1 mequiv) DDQ (as described).[13a] The rust-colored desalted and dried raw product was then dissolved in 8 mL of MeOH, diluted with 32 mL of 100 mm aq. potassium phosphate (pH 7.0), and applied to a C-18 column. The loaded column was first washed with 100 mL 100 mm aq. potassium phosphate (pH 7.0)/MeOH 8:2 (v/v). The solvent composition was linearly changed within 30 min to buffer/MeOH 4:6 (v/v), allowing for the collection of an NCC-containing fraction. MeOH was removed at a rotary evaporator leaving a slightly yellow solution, which was loaded onto a pre-conditioned 5 g *SepPak* cartridge. After washing with 100 mL of water, the raw NCC mixture was eluted with 50 mL of MeOH, and dried in vacuo, giving 121.3 mg of a solid residue. This was dissolved in 9 mL of MeOH, diluted with 11 mL 100 mm aq. potassium phosphate buffer (pH 7.0), and applied to an MPLC column. Using 100 mm aq. potassium phosphate (pH 7.0)/MeOH 55:45 (v/v) three major NCC-containing fractions were separated. These were collected and desalted on a pre-conditioned SepPak cartridge as described above. After removal of solvents at 30 mbar the slightly yellow residues were dried in vacuo, giving 18.7 mg (28.3 μmol, 9.1 %) of **6*epi*** and 42.6 mg (63.1 μmol, 20.2 %) of **5*epi***, besides 42.8 mg (66.4 μmol, 21.3 %) of re-isolated **4*epi***. The samples were characterized by spectroscopic means and were used for identification of samples from other experiments by HPLC-co-injection experiments (as described below).

#### Spectroanalytical data

*1-Formyl-3^2^-hydroxy-15-methoxy-19-oxo-16epi-16,19-dihydrophyllobilane* (**5*epi***): UV/Vis (*c*=2.1×10^−5^ m): *λ*_max_ (log*ε*)=315 (4.2), 240 nm (4.3); CD (*c*=6.3×10^−5^ m): *λ*=312 (2.0), 281 (−11.9), 257 (−2.0), 228 nm (28.1); ^1^H NMR (500 MHz, 25 °C, CD_3_OD): *δ*=1.42 (s, H_3_C17^1^), 2.01 (s, H_3_C13^1^), 2.14 (s, H_3_C7^1^), 2.29 (s, H_3_C2^1^), 2.36 (m, H_2_C12^2^), 2.65 (m, H_2_C3^1^), 2.68 (m, H_A_C12^1^), 2.78 (m, H_B_C12^1^), 3.07 (s, H_3_C15^2^), 3.58 (m, H_2_C3^2^), 3.77 (s, H_3_C8^5^), 3.88/4.28 (AB-system, *J*=9.1, HC15 and HC16), 3.96 (s, H_2_C5), 4.97 (s, HC10), 5.35 (dd, *J*=2.3/11.7, H_A_C18^2^), 6.12 (dd, *J*=2.3/17.8, H_B_C18^2^), 6.39 (dd, *J*=11.7/17.8, HC18^1^), 9.40 ppm (s, HC20); ^13^C NMR (500 MHz, 25 °C, CD_3_OD): *δ*=7.7 (2^1^), 8.0 (7^1^), 8.0 (13^1^), 11.1 (17^1^), 21.2 (12^1^), 22.4 (5), 28.6 (3^1^), 36.1 (10), 39.7 (12^2^), 51.7 (8^5^), 54.5 (15^2^), 62.2 (3^2^), 64.7 (16), 67.7 (8^2^), 79.0 (15), 112.6 (7), 118.1 (13), 119.9 (18^2^), 120.1 (3), 121.9 (12), 123.8 (14), 125.6 (8), 125.7 (11), 125.8 (18^1^), 129.4 (18), 129.6 (1), 134.9 (2), 134.9 (6), 138.7 (4), 153.5 (17), 161.0 (9), 171.3 (8^3^), 174.2 (19), 181.6 ppm (12^3^). ESI-MS: *m*/*z* (%): 735.4 (14) [*M*−H+K+Na]^+^, 719.4 (34) [*M*−H+2Na]^+^, 697.4 (100) [*M*+Na]^+^.

*1-Formyl-3^2^,15-dihydroxy-19-oxo-16epi-16,19-dihydrophyllobilane* (**6*epi***): UV/Vis (*c*=2.9×10^−5^ m): *λ*_max_ (log*ε*)=316 (4.2), 243 nm (4.2); CD (*c*=6.2×10^−5^ m): *λ*=312 (2.0), 280 (−12.0), 255 (−1.2), 228 nm (28.1); ^1^H NMR (500 MHz, 25 °C, CD_3_OD): *δ*=1.55 (s, H_3_C17^1^), 2.01 (s, H_3_C13^1^), 2.12 (s, H_3_C7^1^), 2.29 (s, H_3_C2^1^), 2.35 (m, H_2_C12^2^), 2.65 (m, H_2_C3^1^), 2.68 (m, H_A_C12^1^), 2.78 (m, H_B_C12^1^), 3.58 (m, H_2_C3^2^), 3.76 (s, H_3_C8^5^), 3.96 (s, H_2_C5), 4.26/4.42 (AB-system, *J*=7.6, HC16 and HC15), 4.94 (s, HC10), 5.36 (dd, *J*=2.4/11.7, H_A_C18^2^), 6.12 (dd, *J*=2.4/17.8, H_B_C18^2^), 6.41 (dd, *J*=11.7/17.8, HC18^1^), 9.40 ppm (s, HC20); ^13^C NMR (500 MHz, 25 °C, CD_3_OD): *δ*=9.0 (2^1^), 9.0 (7^1^), 9.0 (13^1^), 11.5 (17^1^), 21.7 (12^1^), 23.5 (5), 27.4 (3^1^), 36.6 (10), 40.2 (12^2^), 52.3 (8^5^), 62.6 (3^2^), 65.3 (16), 68.1 (8^2^), 68.9 (15), 112.0 (7), 116.1 (13), 119.5 (18^2^), 121.3 (3), 121.5 (12), 125.6 (11), 125.7 (8), 126.6 (18^1^), 127.9 (14), 129.7 (1), 130.7 (18), 134.5 (6), 134.9 (2), 139.1 (4), 154.9 (17), 161.5 (9), 171.5 (8^3^), 175.5 (19), 181.5 ppm (12^3^); ESI-MS: *m*/*z* (%): 737.4 (14) [*M*−H+2K]^+^, 721.4 (67) [*M*−H+K+Na]^+^, 699.4 (100) [*M*+K]^+^, 683.4 (72) [*M*+Na]^+^, 560.3 (10) [*M*−ring D+Na]^+^.

**HPLC-analytical comparison of 5*epi* and 6*epi* derived from oxidation of 4*epi* with**­ ***Spathiphyllum wallisii***­ **leaf extracts with their analogues from DDQ oxidation**: The *epi*NCC samples **6*epi***, obtained from oxidation of **4*epi*** in extracts of *Sp. wallisii* leaves, or via chemical oxidation with DDQ (see above), were identified by HPL-chromatograpy. Solutions of the two purified NCC-samples were separately applied to analytical HPLC, as well as in a 1:1 mixture (v/v) of both of them, showing the same elution properties for the NCC from both sources (see Figure S7).

In the same way, *epi*NCC samples **5*epi***, obtained from oxidation of **4*epi*** in extracts of *Sp. wallisii* leaves, or from DDQ oxidation, were compared and identified by UV and mass spectra, as well as HPL-chromatographic comparison with authentic **5*epi*** (see Figure S7).

**Conversion of 6*epi* to analogues in acidic buffer/methanolic solutions**: A 3.5×10^−5^ m solution of **6*epi*** in 100 mm aq. buffer/MeOH 7:3 (v/v) was stored at different pH values (for pH 3.0, 4.0 and 5.0: ammonium acetate/acidic acid buffer; for pH 6.0: potassium phosphate buffer) at room temperature in the dark. After 5 h 200 μL of the solutions were applied to HPLC and (in case of the experiment at pH 3) to LC-MS, showing pH dependent formation of two 15-OMe-NCCs as well as elimination reactions to YCC **7** (from retention time, UV/Vis spectra and mass spectrometry, see Figure 4).

**Preparative conversion of 15-OH NCC 6*epi* to its 15-OMe analogues, 5*epi* and**­ ***iso*****-5*epi* in acidic buffer/methanolic solutions**: About 3 mg of **6*epi*** (synthesis and isolation see above, concentration in solution 3.5×10^−5^ m) were stirred in 100 mm ammonium acetate/acidic acid buffer (pH 3.0)/MeOH 7:3 (v/v) at room temperature under Ar in the dark (see Figure 4). After 5 h, the reaction mixture was diluted with 200 mL of 50 mm phosphate buffer (pH 7.0) and applied to a pre-conditioned 5 g SepPak cartridge. After washing with 150 mL of H_2_O, the raw product was eluted with MeOH and dried under reduced pressure. The obtained residue was dissolved in 50 mm phosphate buffer (pH 7.0)/MeOH 8:2 (v/v) and purified by MPLC. The catabolite ***iso*****-5*epi*** was isolated with 50 mm phosphate buffer (pH 7.0)/MeOH 40:60 (v/v) as solvent mixture. The NCC containing fraction was diluted with the same volume of 50 mm phosphate buffer (pH 7) and applied to a 5 g SepPak cartridge. After washing with 150 mL of H_2_O, the product was eluted with MeOH and dried under reduced pressure. The obtained residue was precipitated in MeOH (50 μL)/CH_2_Cl_2_ (1.8 mL)/*n*-hexane (2 mL) and used for the following analyses.

#### Spectroanalytical data

*1-Formyl-3^2^-hydroxy-15-methoxy-19-oxo-15epi,16epi-16,19-dihydro-phyllobilane (**iso*****-5*epi***): UV/Vis (online, 50 mm aq. potassium phosphate (pH 7.0)/MeOH about 40:60 (v/v)): *λ*_max_ (*ε*_rel_)=314 (1.00), 241 nm (sh, 1.22); CD (*c*=3.1×10^−5^ m): *λ*=314 (1.0), 280 (−6.0), 254 (−1.9), 248 (−2.3), 224 nm (9.4); ^1^H NMR (500 MHz, 25 °C, CD_3_OD): *δ*=1.97 (s, H_3_C13^1^), 2.08 (s, H_3_C7^1^), 2.11 (s, H_3_C17^1^), 2.24 (s, H_3_C2^1^), 2.26 (m, H_A_C12^2^), 2.31 (m, H_B_C12^2^), 2.62 (m, H_2_C3^1^), 2.65 (m, H_A_C12^1^), 2.73 (m, H_B_C12^1^), 3.11 (s, H_3_C15^2^), 3.48 (t, *J*=7.1, H_2_C3^2^), 3.75 (s, H_3_C8^5^), 3.96 (s, H_2_C5), 4.17/4.19 (AB-system, *J*=7.0, HC16 and HC15), 4.92 (s, HC10), 5.34 (dd, *J*=2.3/11.3, H_A_C18^2^), 6.06 (dd, *J*=2.3/17.8, H_B_C18^2^), 6.43 (dd, *J*=11.3/17.8, HC18^1^), 9.32 ppm (s, HC20); ^13^C NMR (500 MHz, CD_3_OD): *δ*=8.8 (2^1^), 9.0 (7^1^), 9.5 (13^1^), 13.7 (17^1^), 22.0 (12^1^), 23.7 (5), 27.9 (3^1^), 37.1 (10), 40.1 (12^2^), 52.7 (8^5^), 56.0 (15^2^), 62.5 (3^2^), 64.3 (16), 67.5 (8^2^), 78.0 (15), 112.1 (7), 118.2 (13), 119.1 (18^2^), 120.8 (3), 120.8 (12), 124.5 (14), 125.7 (8), 125.9 (11), 126.8 (18^1^), 129.1 (1), 129.3 (18), 133.9 (6), 135.1 (2), 138.1 (4), 155.7 (17), 160.8 (9), 171.4 (8^3^), 174.7 (19), 181.9 ppm (12^3^). LC/ESI-MS: *m*/*z* (%): 751.1 (56) [*M*−H+2K]^+^, 735.2 (14) [*M*−H+K+Na]^+^, 713.3 (48) [*M*+K]^+^, 697.1 (13) [*M*+Na]^+^, 677.1 (11), 676.1 (37), 675.1 (100) C_36_H_43_N_4_O_9_^+^, [*M*+H]^+^, 643.2 (63) [*M*−CH_3_OH+H]^+^, 611.3 (13) [*M*−2CH_3_OH+H]^+^, 552.2 (65) [*M*−ring D+H]^+^, 520.2 (14) [*M*−ring D−CH_3_OH+H]^+^.

### Formation of YCC 7 from 15-hydroxy-NCC 6*epi* by water elimination under acidic conditions**:**

A 4.5×10^−5^ m solution of **6*epi*** in 100 mm aq. potassium phosphate (pH 3.0, 4.0 and 5.0)/ACN 7:3 (v/v) was stored in the dark at room temperature. Over the time course of 14 h (22 h for the experiment at pH 5.0) UV/Vis spectra were acquired to measure formation of **7** (via its absorption band at 426 nm). After 4 h at pH 3.0 more than 95 % of **6*epi*** were converted, after the same time at pH 4.0 almost the half of **6*epi*** and at pH 5.0 at least 16 % of **6*epi*** were transformed (see Figure 5). The reactions were analyzed to have reached 50 % conversion at 35 min for pH 3.0, 227 min for pH 4.0 and 1215 min for pH 5.0. Calculated pseudo-first order rate constants *k* (pH 3)=1.19 h^−1^, *k* (pH 4)=0.18 h^−1^ and *k* (pH 5)=0.034 h^−1^. For the calculations of rates, the extinction coefficient of the NCC **4*epi*** was used, as well as that of YCC **7**. Data were normalized with respect to the turnover achieved at pH 3.

### HPLC analyses of Chl-catabolites in senescent *Spathiphyllum wallisii* leaves, varying incubation and extraction procedures**:**

*Basic procedure*: A yellow-greenish senescent leaf part (3 cm^2^, about 200 mg wet weight, *Spathiphyllum wallisii*) was ground with sea sand in a mortar at ambient temperature. After extraction with 400 μL of MeOH and centrifugation of the resulting suspension for 5 min at 13 000 rpm, the supernatant was diluted 1:1 (v/v) with 50 mm aq. potassium phosphate (pH 7.0) and centrifuged (5 min, 13 000 rpm) once more. The resulting yellow extract was analyzed by (reversed phase) HPLC, using absorbance and fluorescence detection. Besides the fluorescent chlorophyll catabolite *Sw*-FCC-62 (**3**), three more polar NCC fractions **4**, **5** and **6** were observed, as was the less polar fraction of the YCC **7** (see Figure 1).

The basic incubation and extraction procedure (see above) was varied, using analytical HPLC, to study the presence of **4**, **5** and **6**, as follows:

*Effect of temperature*: The formation of the oxidized derivatives **5** and **6** was found to be insignificant, when plant material and mortar were cooled with liquid nitrogen during the extraction (see Figure 1).

*Absence of methanol in the extract*: Incubation of ground lyophilized plant material for five minutes with water (at ambient temperature in the dark) led to formation of **6** (and **5** was only hardly detected). The same result was observed, when ground (water containing) fresh leaf parts were stored in the dark for five minutes, before HPLC-analysis. When acetone was used for extraction, instead of methanol, again **6** (but no **5**) was observed (see Supporting Information, Figure S11).

*Relevance of basic components:* When ground senescent *Sp. wallisii* leaf parts were centrifuged, the pellet separated from supernatant, and **4*epi*** was added to both separated fractions, formation of **5*epi*** and **6*epi*** was strongly diminished. When the ground plant material was heated at 70 °C for 10 min before addition of **4*epi***, **5*epi*** and **6*epi*** were not observed (see Supporting Information, Figure S12). When, the ground plant material was evacuated by applying high vacuum for 20 seconds, and a deoxygenated solution of **4*epi*** was added under N_2_-protection, oxidation of **4*epi*** was insignificant after 30 min storage. Likewise, oxidation of **4*epi*** was insignificant, when a previously deoxygenated mixture was stored for 30 min in an atmosphere of CO/air 1:1 (v/v). In contrast, storage of the mixture under O_2_ led to increased formation of **5*epi*** and **6*epi*** (see Figure 6).

Effect of added cyanide or hydroxide: Likewise, formation of the oxidized derivatives **5** and **6** was found to be insignificant when ≥3 mm aq. KCN or the same amount of 3 mm KOH were added to the senescent leaf material, before grinding it (at room temperature) followed by HPLC analysis (as above and Supporting Information, Figure S10).

## Supporting Information

As a service to our authors and readers, this journal provides supporting information supplied by the authors. Such materials are peer reviewed and may be re-organized for online delivery, but are not copy-edited or typeset. Technical support issues arising from supporting information (other than missing files) should be addressed to the authors.

miscellaneous_information

## References

[1a] Hörtensteiner S, Lee DW, Gan S (2007). Senescence Processes in Plants.

[1b] Kräutler B (2008). Photochem. Photobiol. Sci.

[2] Moser S, Müller T, Oberhuber M, Kräutler B (2009). Eur. J. Org. Chem.

[3a] Kräutler B, Hörtensteiner S, Ferreira GC, Kadish KM, Smith KM, Guilard R (2013). Chlorophyll Breakdown - Chemistry, Biochemistry and Biology, Vol. 28.

[3b] Kräutler B (2014). Chem. Soc. Rev.

[4] Kräutler B, Jaun B, Bortlik K, Schellenberg M, Matile P (1991). Angew. Chem.

[b01] (1991). Angew. Chem. Int. Ed. Engl.

[5] Hörtensteiner S, Kräutler B (2011). Biochim. Biophys. Acta Bioenergetics.

[6a] Müller T, Rafelsberger M, Vergeiner C, Kräutler B (2011). Angew. Chem.

[b02] (2011). Angew. Chem. Int. Ed.

[6b] Christ B, Süssenbacher I, Moser S, Bichsel N, Egert A, Müller T, Kräutler B, Hörtensteiner S (2013). Plant Cell.

[7] Oberhuber M, Berghold J, Breuker K, Hörtensteiner S, Kräutler B (2003). Proc. Natl. Acad. Sci. USA.

[8] Banala S, Moser S, Müller T, Kreutz C, Holzinger A, Lütz C, Kräutler B (2010). Angew. Chem.

[b03] (2010). Angew. Chem. Int. Ed.

[9] Kräutler B, Banala S, Moser S, Vergeiner C, Müller T, Lütz C, Holzinger A (2010). FEBS Lett.

[10] Moser S, Müller T, Holzinger A, Lütz C, Jockusch S, Turro NJ, Kräutler B (2009). Proc. Natl. Acad. Sci. USA.

[11] Jockusch S, Turro NJ, Banala S, Kräutler B (2014). Photochem. Photobiol. Sci.

[12] Moser S, Müller T, Ebert M-O, Jockusch S, Turro NJ, Kräutler B (2008). Angew. Chem.

[b04] (2008). Angew. Chem. Int. Ed.

[13a] Moser S, Ulrich M, Müller T, Kräutler B (2008). Photochem. Photobiol. Sci.

[13b] Ulrich M, Moser S, Müller T, Kräutler B (2011). Chem. Eur. J.

[14a] Scherl M, Müller T, Kräutler B (2012). Chem. Biodiversity.

[14b] Wakana D, Kato H, Momose T, Sasaki N, Ozeki Y, Goda Y (2014). Tetrahedron Lett.

[14c] Moser S, Müller T, Holzinger A, Lütz C, Kräutler B (2012). Chem. Eur. J.

[15] Müller T, Ulrich M, Ongania K-H, Kräutler B (2007). Angew. Chem.

[b05] (2007). Angew. Chem. Int. Ed.

[16] Curty C, Engel N (1996). Phytochemistry.

[17] Li C, Ulrich M, Liu X, Wurst K, Müller T, Kräutler B (2014). Chem. Sci.

[18] Losey FG, Engel N (2001). J. Biol. Chem.

[19] Frankenberg N, Lagarias JC, Kadish KM, Smith KM, Guilard R (2003). Handbook of Porphyrin Sciences, Vol. 13.

[20a] Ernst L (1981). Liebigs Ann. Chem.

[20b] Kessler H, Gehrke M, Griesinger C (1988). Angew. Chem.

[b06] (1988). Angew. Chem. Int. Ed. Engl.

[21] Gottlieb HE, Kotlyar V, Nudelman A (1997). J. Org. Chem.

[22a] Fenn JB, Mann M, Meng CK, Wong SF, Whitehouse CM (1989). Science.

[22b] Müller T, Vergeiner S, Kräutler B (2014). Int. J. Mass Spectrom.

