# Supplementary material for: Stereo- and Regioselective Phyllobilane Oxidation in Leaf Homogenates of the Peace Lily (Spathiphyllum wallisii): Hypothetical Endogenous Path to Yellow Chlorophyll Catabolites
Source: Chemistry. 2014 Nov 7;21(1):136–49. doi: 10.1002/chem.201404783 (PMC4517098; doi:10.1002/chem.201404783)
Supplement: Supplementary file 1 — miscellaneous_information [file chem0021-0136-sd1.pdf]

CHEMISTRY
A European Journal

Supporting Information

© Copyright Wiley-VCH Verlag GmbH & Co. KGaA, 69451 Weinheim, 2014

Stereo- and Regioselective Phyllobilane Oxidation in Leaf Homogenates of the Peace Lily (*Spathiphyllum wallisii*): Hypothetical Endogenous Path to Yellow Chlorophyll Catabolites

Clemens Vergeiner, Markus Ulrich, Chengjie Li, Xiujun Liu, Thomas Müller, and Bernhard Kräutler*[a]

chem_201404783_sm_miscellaneous_information.pdf

Manuscript Chem.201404783

Supporting Information

Methods:

Analytical HPLC: Shimadzu HPLC system, used with manual sampler, *DGU-20A5* online degasser, *LC-20AD* pump, *CBM-20A* system controller, *SPD-M20A* diode array detector, *Jasco FP-920* fluorescence detector, a *Rheodyne* injection valve with 20 or 200 µL loop. Data were collected and processed with *Shimadzu LC Solution*. *Phenomenex Hyperclone* ODS 5 µm 250 x 4.6 mm i.d. colomn connected to a *Phenomenex* ODS 4 x 3 mm i.d. pre-colomn used at room temperature with a flow rate of 0.5 $mL.min^{-1}$. Solvent system: solvent A: 50 mM aq. potassium phosphate (pH 7.0), solvent B: MeOH; solvent composition (A/B) as function of time: analytical and co-injection experiments: 0 - 32 min: 55/45 to 39/81; 32 - 37 min: 39/81 to 0/100; 37 - 45 min: 0/100; 45 - 50 min: 0/100 to 55/45; analytical experiments with ***4epi***: 0 - 22 min: 55/45 to 44/56; 22 - 25 min: 44/56 to 0/100; 25 - 30 min: 0/100; 30 - 35 min: 0/100 to 55/45 or: 0 - 5 min: 80/20; 5 – 55 min: 80/20 to 30/70; 55 - 60 min: 30/70 to 0/100; 60 - 70 min: 0/100; 70 - 75 min: 0/100 to 80/20;

LC/ESI-MS: LC Packings Ultimate HPLC system, used with manual sampler and helium degasser. *UVD340U* diode array detector and a *Rheodyne* injection valve with 30 µL loop. Data were collected and processed with *Chromeleon V6.50*. *Phenomenex Hyperclone* ODS 5 µm 250 x 4.6 mm i.d. colomn connected to a *Phenomenex* ODS 4 x 3 mm i.d. pre-colomn used at room temperature with a flow rate of 0.5 $mL.min^{-1}$. Solvent system: solvent A: 10 mM aq. ammonium acetate, solvent B: MeOH; solvent composition (A/B) as function of time: 0 - 85 min: 65/35 to 32/68; 85 - 88 min: 32/68 to 0/100; 88 - 96 min: 0/100; 96 - 100 min: 0/100 to 65/35. Coupled with *Finnigan MAT 95-S* or *Finnigan LCQ Classic* mass spectrometer (conditions see below).

Spectroscopy:

Ultraviolet/visible (UV/Vis): λ_{max} [nm] (log ε or ε_{rel}), *Hitachi U-3000* spectrophotometer, solvents: MeOH or (online) HPLC elution mixtures; concentrations of NCCs were calculated using the published extinction coefficients of NCC ***4epi*** at 312 nm (log ε = 4.23 [1]) and of YCC **7** (*Cj*-YCC) at 426 nm (log ε = 4.51 [2]).

Circular dichroism (CD): $\lambda_{min/max}$ [nm] ($\Delta\varepsilon$), *Jasco J715,* solvent: MeOH.

Nuclear magnetic resonance (NMR): δ [ppm], J [Hz], *Bruker UltraShield Avance II+* 600 MHz or *Varian Unity Inova* 500 MHz; $^{1}H,^{1}H$-homonuclear (COSY, ROESY) and $^{1}H,^{13}C$-heteronuclear (HSQC, HMBC) experiments;[3] 10 °C or 25 °C; residual solvent peaks (CD_2HOD: δ_H = 3.31 ppm, δ_C = 49.00 ppm; CD_2HCN: δ_H = 1.94 ppm, δ_C = 1.32 ppm) [4] were used as internal references; signals are classified as singlet (s), doublet (d), double doublet (dd), triplet (t) and multiplet (m); broad = br.

Electrospray ionization mass spectrometry (ESI-MS):[5] *m*/*z* (rel. abundance; type of ion); signals due to isotopomers and their relative intensities are shown for base peaks and $[M+H]^+$ pseudo molecular ions; *Finnigan MAT 95-S* or *Finnigan LCQ Classic* mass spectrometer, ESI source; positive ion mode, spray voltage 1.4 (*Finnigan MAT 95*) or 4.5 kV (*Finnigan LCQ Classic*).

NMR-spectral data of NCCs 5*epi* and *iso*-5*epi*:

1-Formyl-3^2-hydroxy-15-methoxy-19-oxo-16epi-16,19-dihydrophyllobilane (**5*epi***): ^{1}H-NMR (500 MHz, 25 °C, $CD_3CN/CDCl_3$ 1/1 (v/v)): 1.37 (s, H_3C17^1), 1.95 (s, H_3C13^1), 2.15 (s, H_3C7^1), 2.16 (s, H_3C2^1), 2.37 (m, H_AC12^2), 2.41 (m, H_BC12^2), 2.61 (m, H_2C3^1), 2.73 (m, H_2C12^1), 3.00 (s, H_3C15^2), 3.44 (m, H_AC3^2), 3.52 (m, H_BC3^2), 3.69 (s, H_3C8^5), 3.75/3.79 (AB-system, *J* = 15.7, H_2C5), 3.84 (d, *J* = 3.3, $HC8^2$), 3.89/3.93 (AB-system, *J* = 7.6, HC15 and HC16), 4.82 (d, *J* = 3.3, HC10), 5.28 (dd, *J* = 2.5/11.5, H_AC18^2), 6.13 (dd, *J* = 2.5/17.6, H_BC18^2), 6.29 (dd, *J* = 11.5/17.6, $HC18^1$), 6.40 (s, HN24), 8.27 (s, HN23), 9.33 (s, HC20), 10.2 (br. s, HN), 12.5 (br. s, HN); ^{13}C-NMR (600 MHz, 25 °C, $CD_3CN/CDCl_3$ 1/1 (v/v)): 9.0 (2^1), 9.1 (13^1), 9.3 (7^1), 11.9 (17^1), 21.0 (12^1), 22.3 (5), 27.2 (3^1), 35.9 (10), 39.5 (12^2), 52.4 (8^5), 55.8 (15^2), 61.9 (3^2), 63.7 (16), 65.7 (8^2), 78.1 (15), 109.7 (7), 117.4 (13), 118.7 (18^2), 119.9 (3), 121.0 (12), 122.5 (14), 123.4 (8), 126.3 (18^1), 128.7 (1), 128.8 (18), 133.6 (6), 137.3 (4), 152.7 (17), 160.0 (9), 170.5 (8^3), 173.1 (19), 179.6 (12^3).

1-Formyl-3^2-hydroxy-15-methoxy-19-oxo-15*epi*,16*epi*-16,19-dihydrophyllobilane (***iso*-5*epi***): ^{1}H-NMR (500 MHz, 25 °C, $CD_3CN/CDCl_3$ 1/1 (v/v)): 1.81 (s, H_3C13^1), 2.06 (s, H_3C2^1), 2.07 (s, H_3C17^1), 2.17 (s, H_3C7^1), 2.20 (m, H_AC12^2), 2.38 (m, H_BC12^2), 2.53 (m, H_AC3^1), 2.60 (m, H_BC3^1), 2.69 (m, H_AC12^1), 2.79 (m, H_BC12^1), 3.18 (s, H_3C15^2), 3.42/3.85 (AB-system, *J* = 9.0, HC16 and HC15), 3.51 (m, H_2C3^2), 3.71 (s, H_3C8^5), 3.73/3.79 (AB-system, *J* = 16, H_2C5), 3.83 (d, *J* = 3.6, $HC8^2$), 4.79 (d, *J* = 3.6, HC10), 5.32 (dd, *J* = 2.4/11.7, H_AC18^2), 5.96 (s, HN24), 6.19 (dd, *J* = 2.4/17.6, H_BC18^2), 6.43 (dd, *J* = 11.7/17.6, $HC18^1$), 8.21 (s, HN23), 9.23 (s, HC20), 9.45 (s, HN22), 12.8 (s, HN21); ^{13}C-NMR (500 MHz, 25 °C, $CD_3CN/CDCl_3$ 1/1 (v/v)): 8.7 (2^1), 9.1 (13^1), 9.2 (7^1), 13.6 (17^1), 20.8 (12^1), 22.5 (5), 26.9 (3^1), 35.8 (10), 40.7 (12^2), 52.4 (8^5), 56.3 (15^2), 61.4 (3^2), 64.2 (16), 65.8 (8^2), 77.7 (15), 111.4 (7), 118.5 (18^2), 119.9 (13), 121.0 (3), 122.1 (12), 125.0 (8), 125.9 (11), 126.3 (14), 126.5 (18^1), 128.6 (18), 129.7 (1), 133.5 (2), 134.6 (6), 137.9 (4), 156.8 (17), 171.6 (8^3), 174.2 (19), 176.2 (20), 182.1 (12^3).

Table S1: ^{1}H- and ^{13}C-NMR chemical shift values of 15-OMe-NCC **5** (600 MHz, 10°C, CD_3OD), 15-OMe-*epi*NCCs **5*epi*** and ***iso*-5*epi*** (both 500 MHZ, 25°C) and of 15-OH-*epi*NCC **6*epi*** (600 MHz, 10°C). Significant chemical shift differences for HC15, and nuclei near it, are highlighted bold.

	**5**	**5*epi***		***iso*-5*epi***		**6*epi***	
	^{1}H NMR	^{1}H NMR	^{13}C NMR	^{1}H NMR	^{13}C NMR	^{1}H NMR	^{13}C NMR
C1			129.6		129.1		129.7
C2			134.9		135.1		134.9
$C2^1$	2.20	2.29	7.7	2.24	8.8	2.29	9.0
C3			120.1		120.8		121.3
$C3^1$	2.59	2.65	28.6	2.62	27.9	2.65	27.4
$C3^2$	3.64	3.58	62.2	3.48	62.5	3.58	62.6
C4			138.7		138.1		139.1
C5	3.92	3.96	22.4	3.96	23.7	3.96	23.5
C6			134.9		133.9		134.5
C7			112.6		112.1		112.0
$C7^1$	2.16	2.14	8.0	2.08	9.0	2.12	9.0
C8			125.6		125.7		125.7
$C8^2$			67.7		67.5		68.1
$C8^3$			171.3		171.4		171.5
$C8^5$	3.76	3.77	51.7	3.75	52.7	3.76	52.3
C9			161.0		160.8		161.5
C10	4.96	4.97	36.1	4.92	37.1	4.94	36.6
C11			125.7		125.9		125.6
C12			121.9		120.8		121.5
$C12^1$	2.65/2.79	2.68/2.78	21.2	2.65/2.73	22.0	2.68/2.78	21.7
$C12^2$	2.34	2.36	39.7	2.26/2.31	40.1	2.35	40.2
$C12^3$			181.6		181.9		181.5
C13			118.1		118.2		116.1
$C13^1$	1.95	2.01	8.0	1.97	9.5	2.01	9.0
C14			**123.8**		**124.5**		**127.9**
C15	**3.78**	**3.88**	**79.0**	**4.19**	**78.0**	**4.42**	**68.9**
$C15^2$	3.12	3.07	54.5	3.11	56.0		-
C16	**4.10**	**4.28**	64.7	**4.17**	64.3	**4.26**	65.3
C17			**153.5**		**155.7**		**154.9**
$C17^1$	**1.06**	**1.42**	**11.1**	**2.11**	**13.7**	**1.55**	**11.5**
C18			129.4		129.3		130.7
$C18^1$	6.37	6.39	125.8	6.43	126.8	6.41	126.6
$C18^2$	5.35/6.12	5.35/6.12	119.9	5.34/6.06	119.1	5.36/6.12	119.5
C19			174.2		174.7		175.5
C20	9.28	9.40		9.32		9.40	

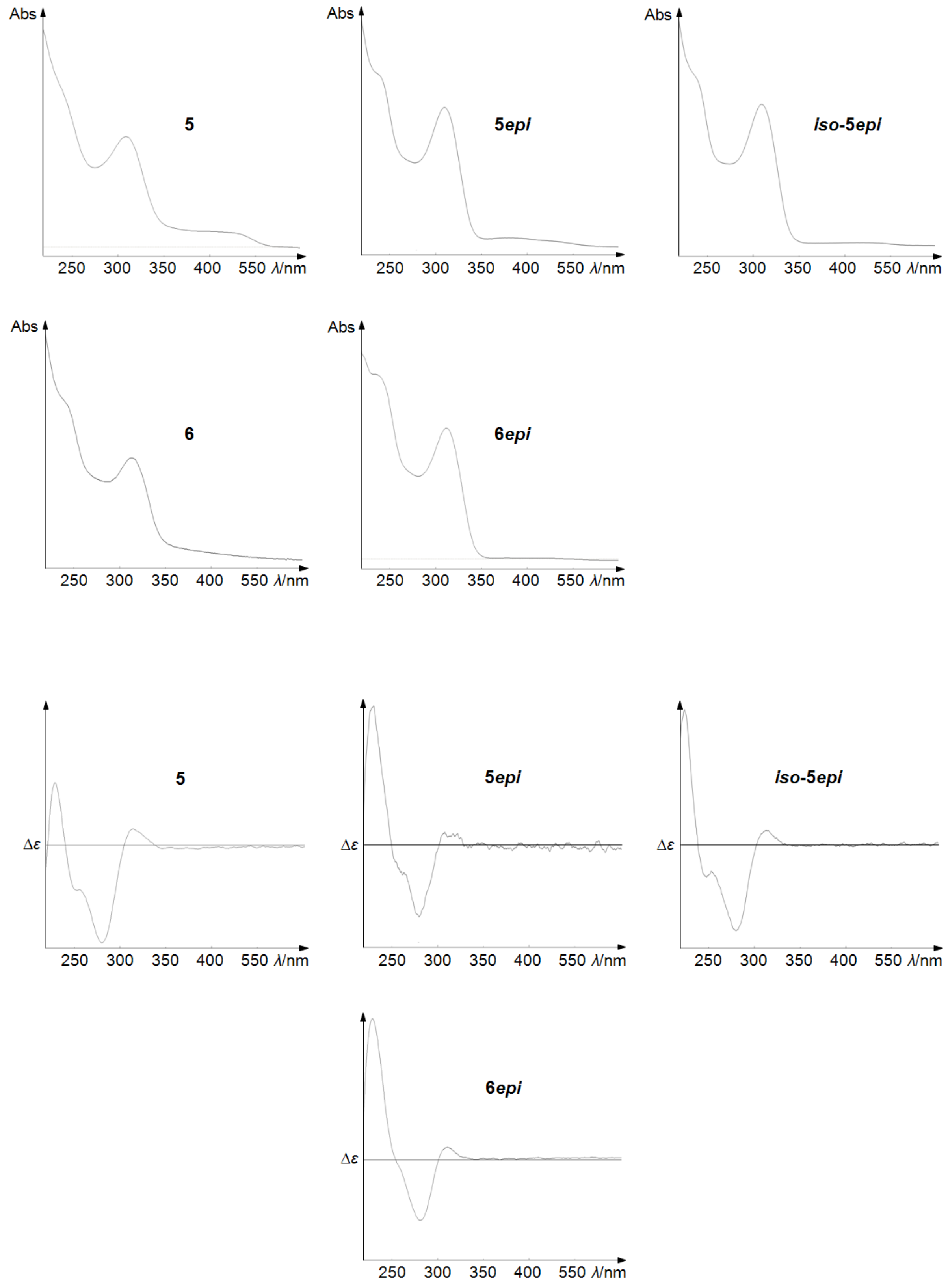

Figure S1: UV/Vis (top) and CD spectra (bottom) of 15-OMe-NCCs **5**, **5*epi*** and ***iso*-5*epi*** and of 15-OH-NCCs **6** and **6*epi***. Spectra were recorded for solutions of NCC **6** in MeOH, or directly online (HPLC).

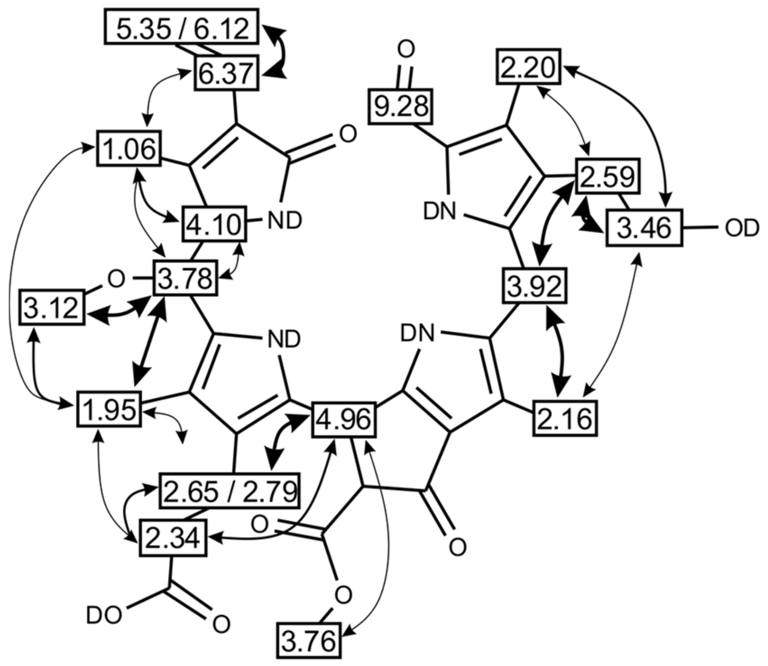

Figure S2: Graphical representation of chemical shift values of 15-OMe-NCC **5** (600 MHz, 10°C CD_3OD), with $^1H,^1H$-ROESY correlations depicted.

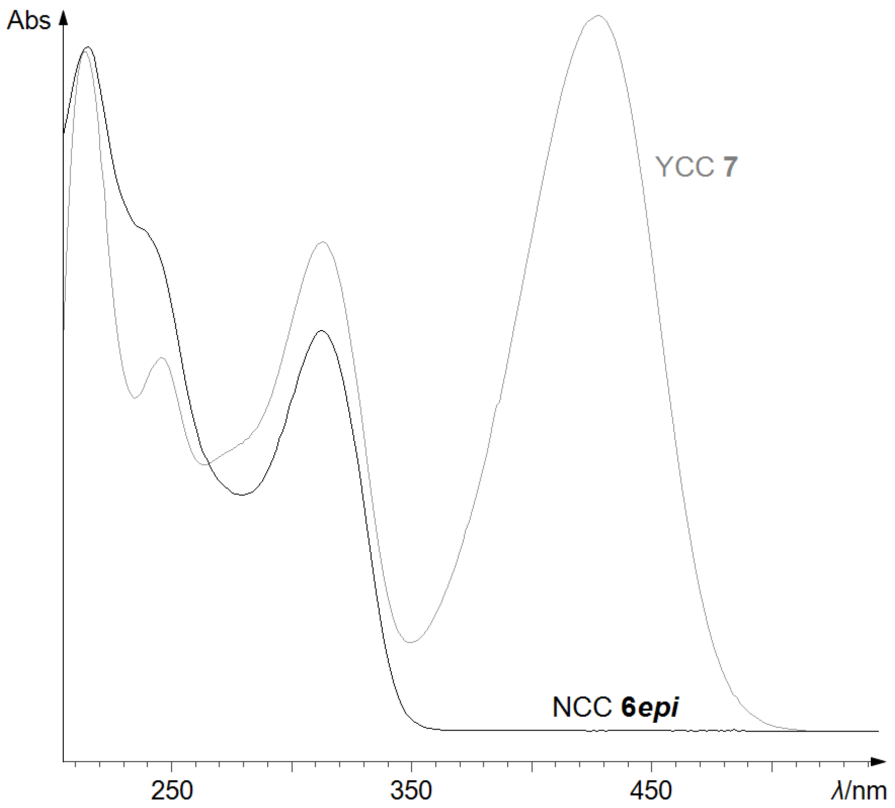

Figure S3: HPLC online UV/Vis spectra of 15-OH-NCC **6*epi*** and of YCC **7**.

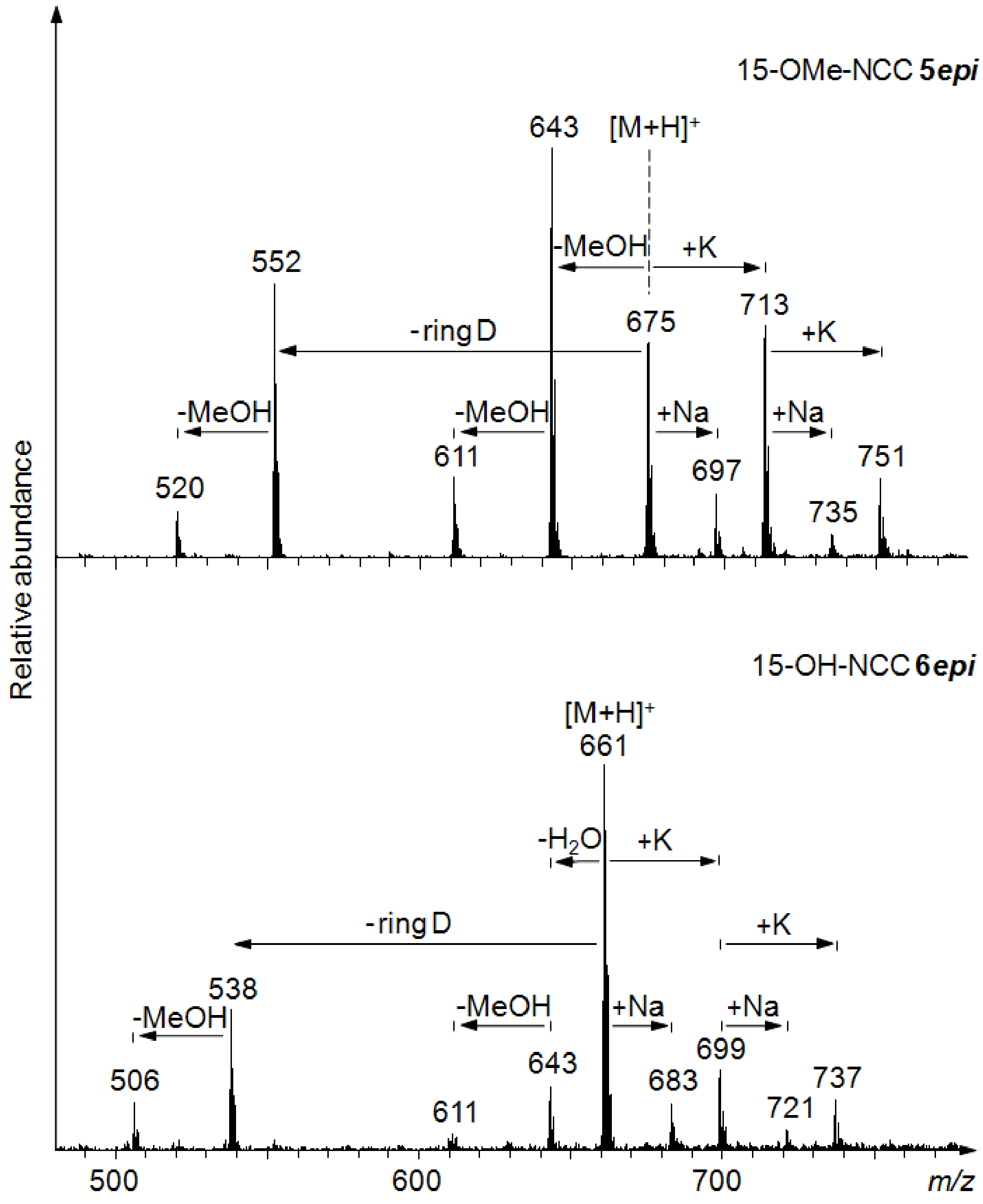

Figure S4: ESI-MS spectra of 15-OMe-NCC **5*epi*** (top) and 15-OH-NCC **6*epi*** (bottom) – see text for details.

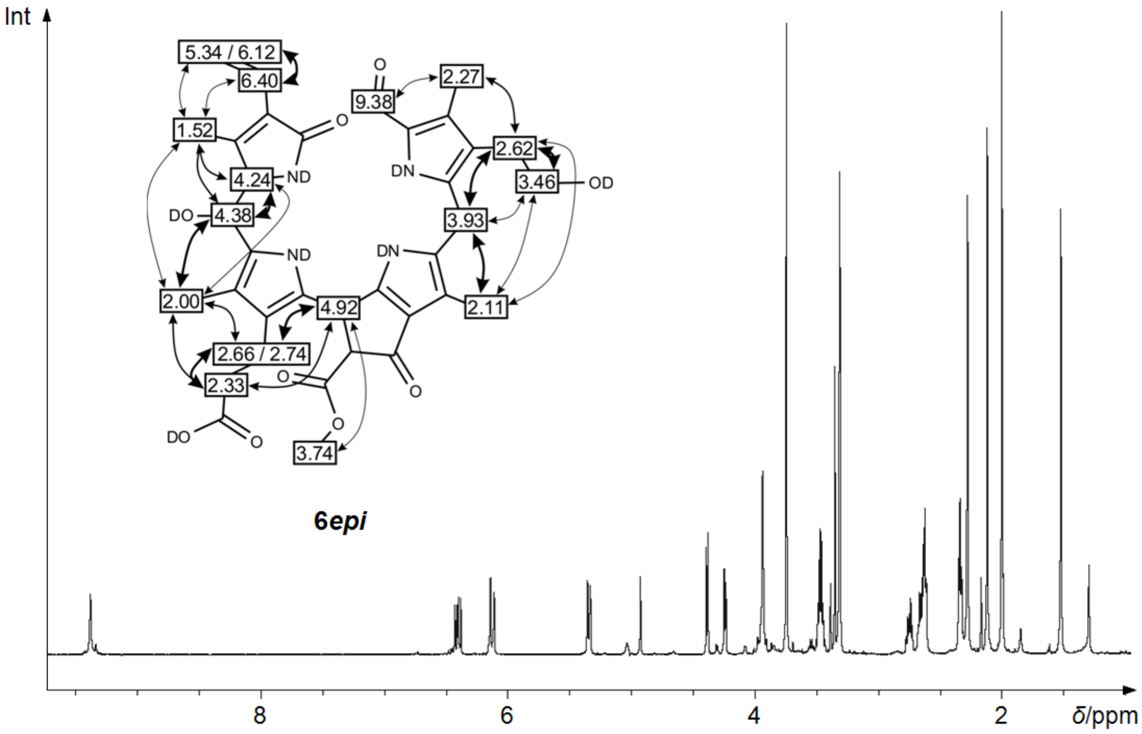

Figure S5: ^{1}H NMR spectrum of 15-OH-NCC ***6epi*** (600 MHz, 10°C, CD_3OD), prepared from a *Sw* leaf homogenate, and graphical representation of chemical shift values with ^{1}H,^{1}H-ROESY correlations.

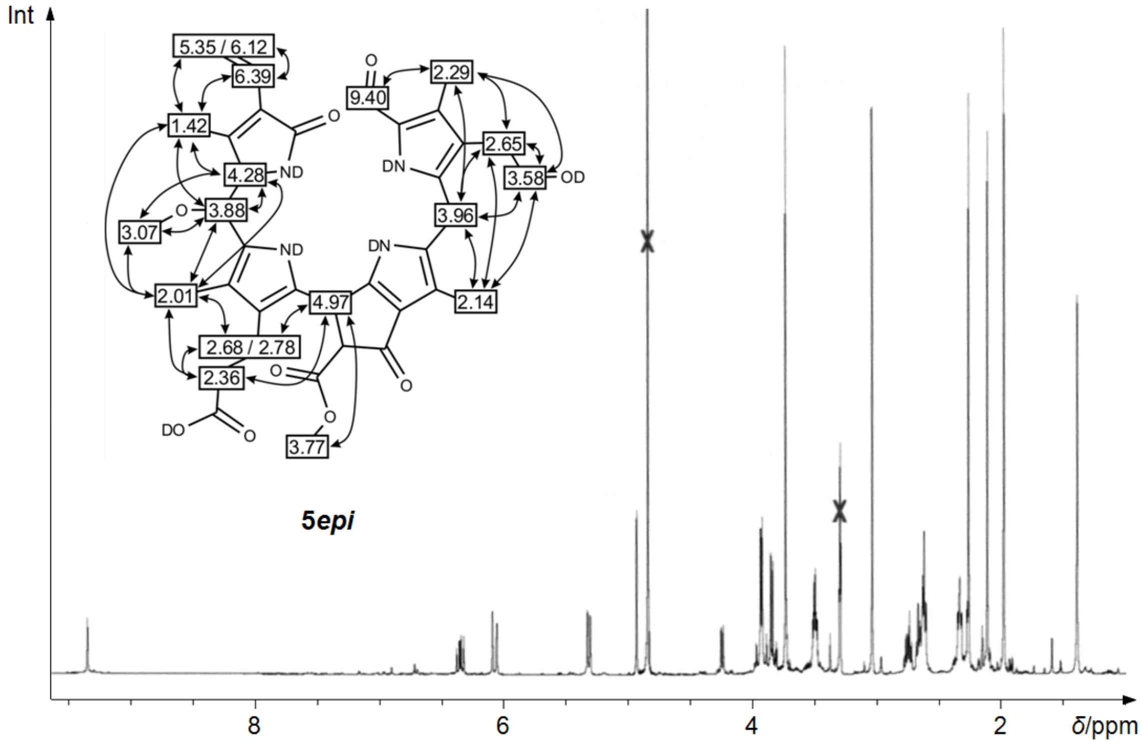

Figure S6: ^{1}H NMR spectrum of 15-OMe-NCC ***5epi*** (500 MHz, 25°C, CD_3OD) and graphical representation of chemical shift values with ^{1}H,^{1}H-ROESY correlations.

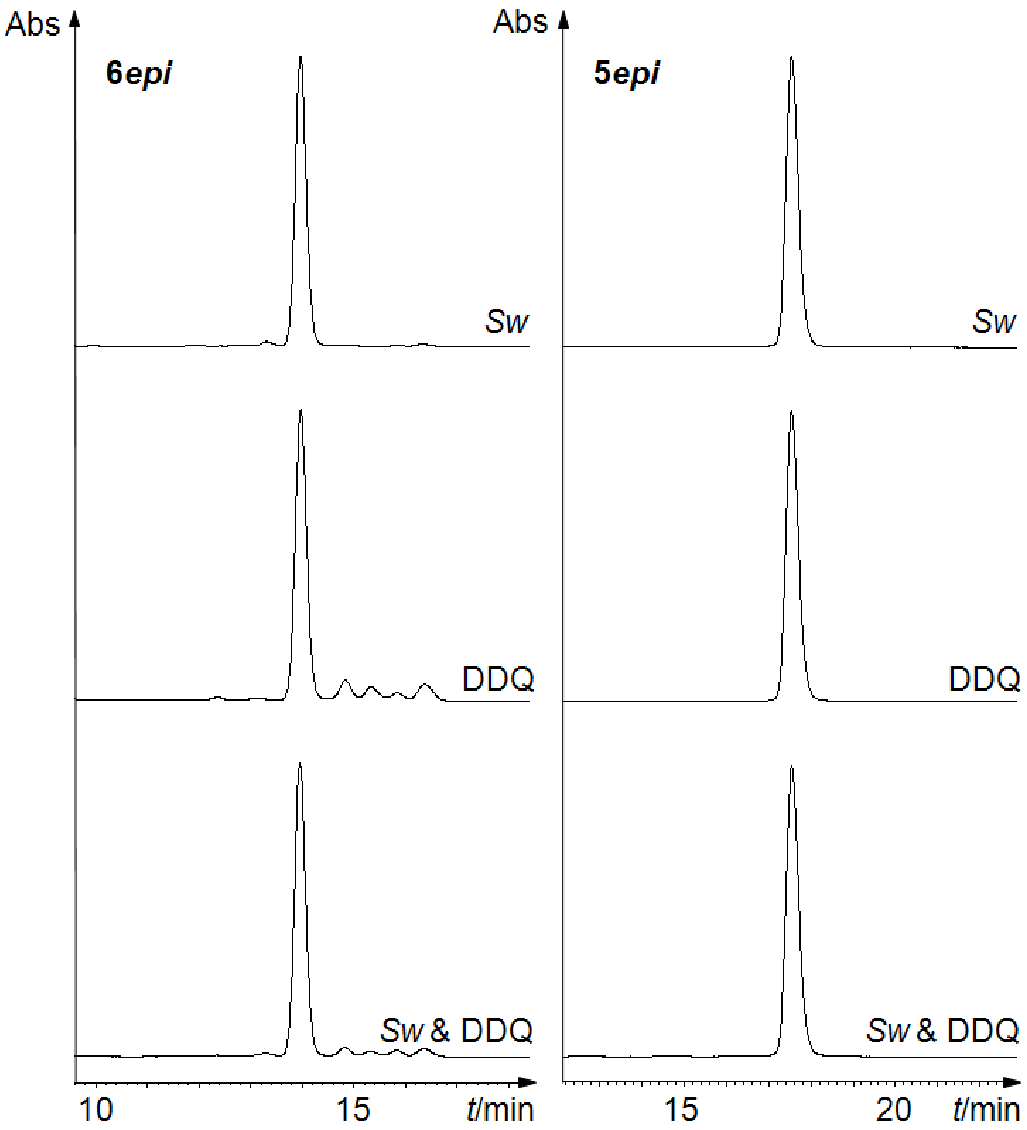

Figure S7: HPLC analyses of **6*epi*** (left) and **5*epi*** (right) prepared with *Sw* leaf homogenates (top) or via oxidation with DDQ (middle) and co-injection experiments of the two samples of NCCs (bottom).

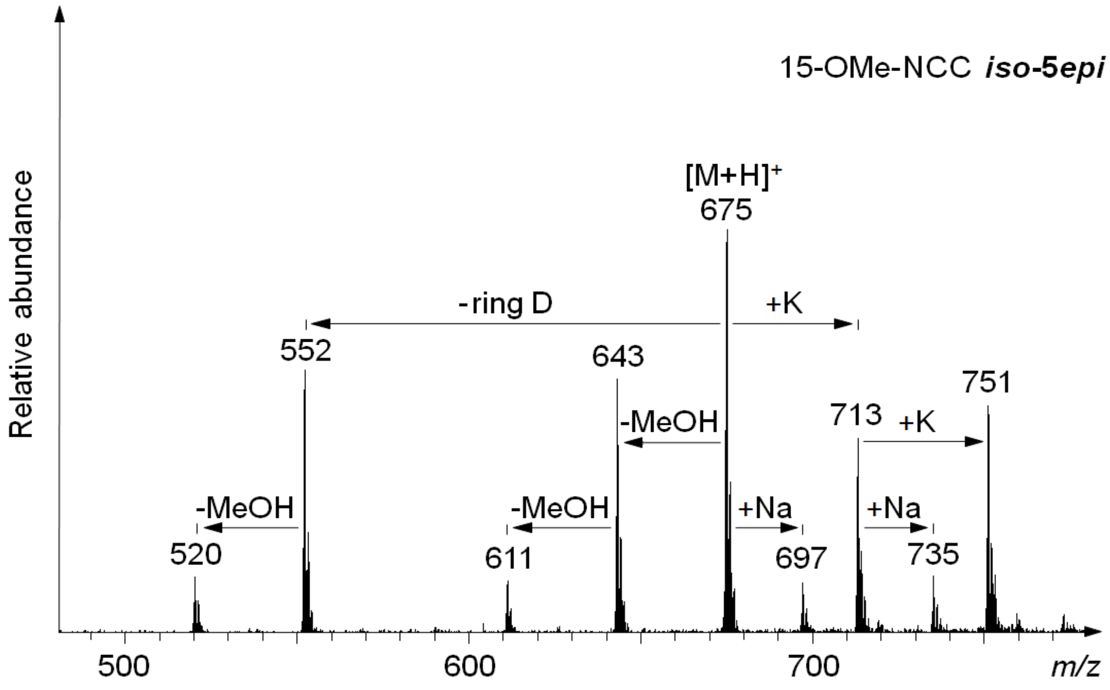

Figure S8: ESI-mass spectrum of the 15-OMe-NCC ***iso*-5*epi*** (see text for details).

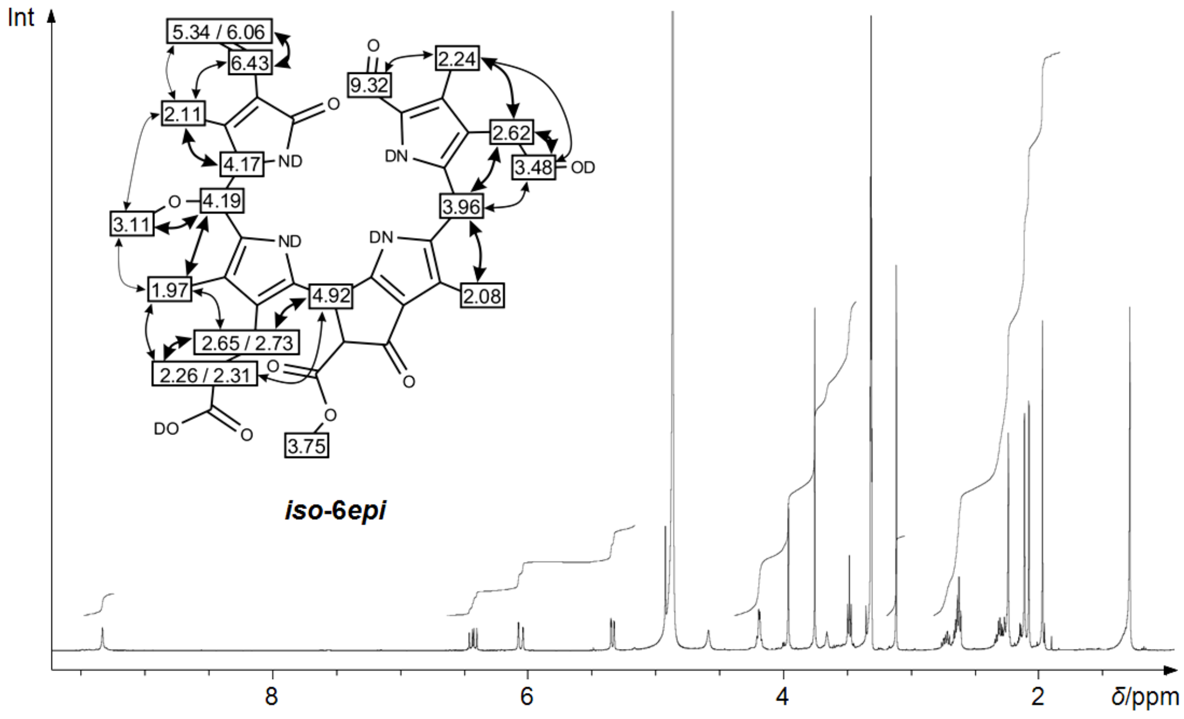

Figure S9: ^{1}H NMR spectrum of 15-OMe-NCC ***iso*-5*epi*** (500 MHz, 25°C, CD_3OD) and graphical representation of chemical shift values with ^{1}H,^{1}H-ROESY correlations.

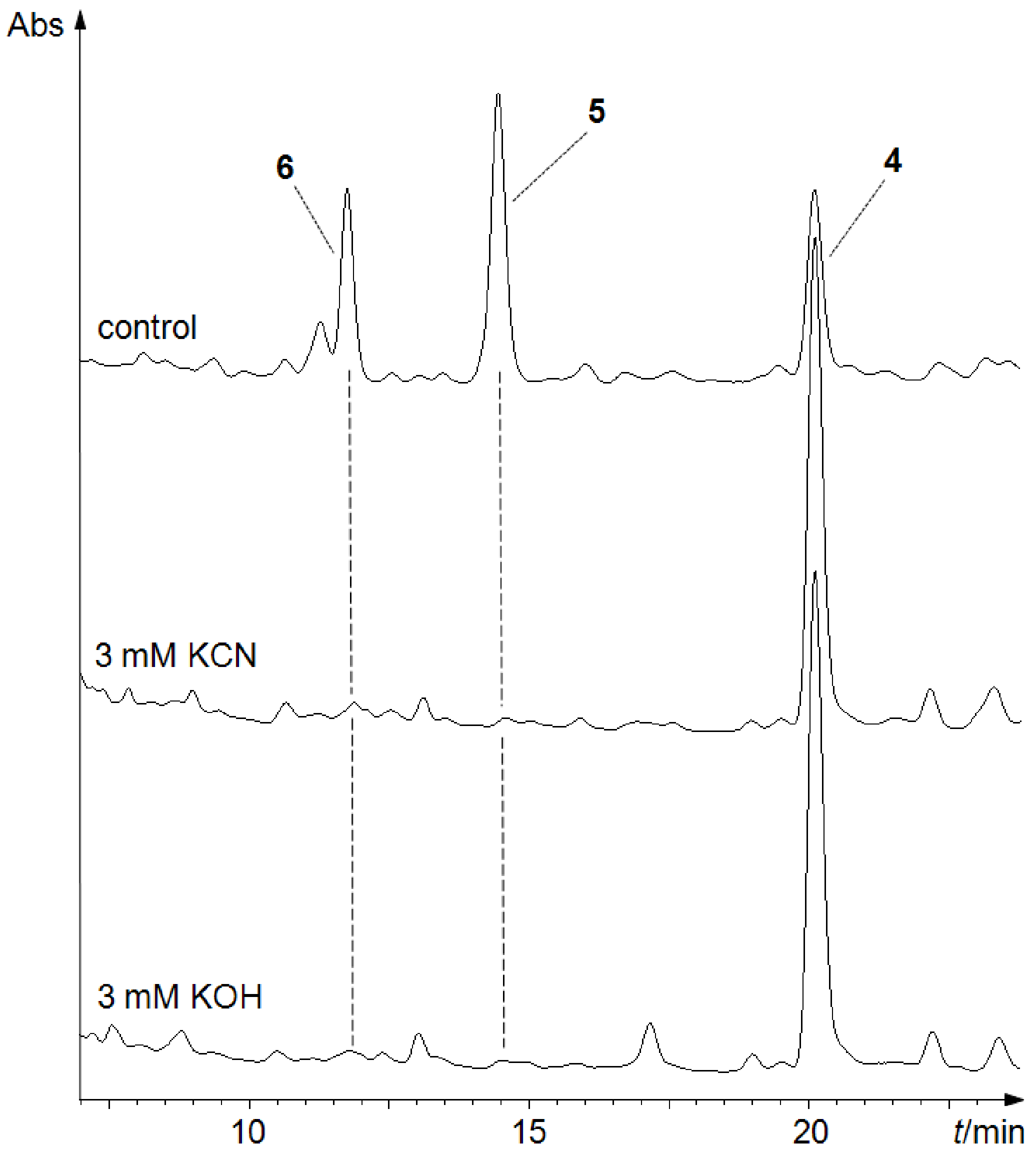

Figure S10: HPLC analyses of methanolic extracts of yellow senescent *Sw* leaf parts (detection at 320 nm). Compared to a control experiment (top, see also Figure 1), addition of 3 mM aq. KCN (middle) or of 3 mM aq. KOH (bottom) inhibited formation of oxidized NCCs **5** and **6** from NCC **4**.

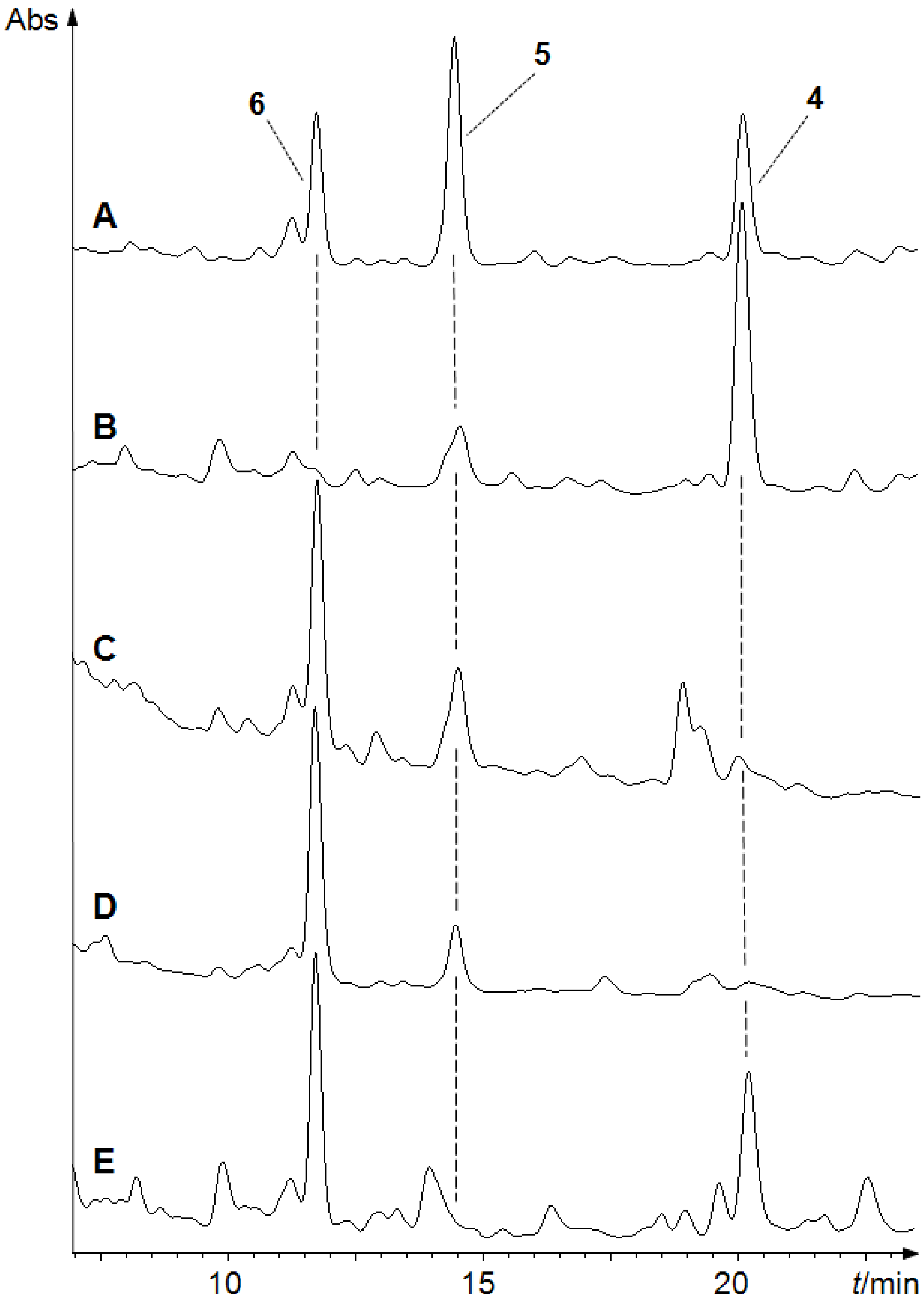

<u>Figure S11:</u> HPLC analyses of methanolic extracts of yellow senescent *Sw* leaf parts (detection at 320 nm). Compared to the control experiment (**A**), lyophilization of the leaf before extraction with MeOH (expt. **B**)furnished strongly diminished formation of 15-OMe-NCC **5** and no detectable 15-OH-NCC **6**, whereas soaking the lyophilized plant material in water for 5 minutes in the dark before extraction (expt. **C**) led to an increase of 15-OH-NCC **6** formation. Similar results were obtained when freshly ground (water containing) leaf parts were kept in the dark for 5 minutes before addition of methanol and further extraction (expt. **D**). Extraction of senescent leaf parts with acetone instead of methanol led to formation of 15-OH-NCC **6** exclusively (expt. **E**).

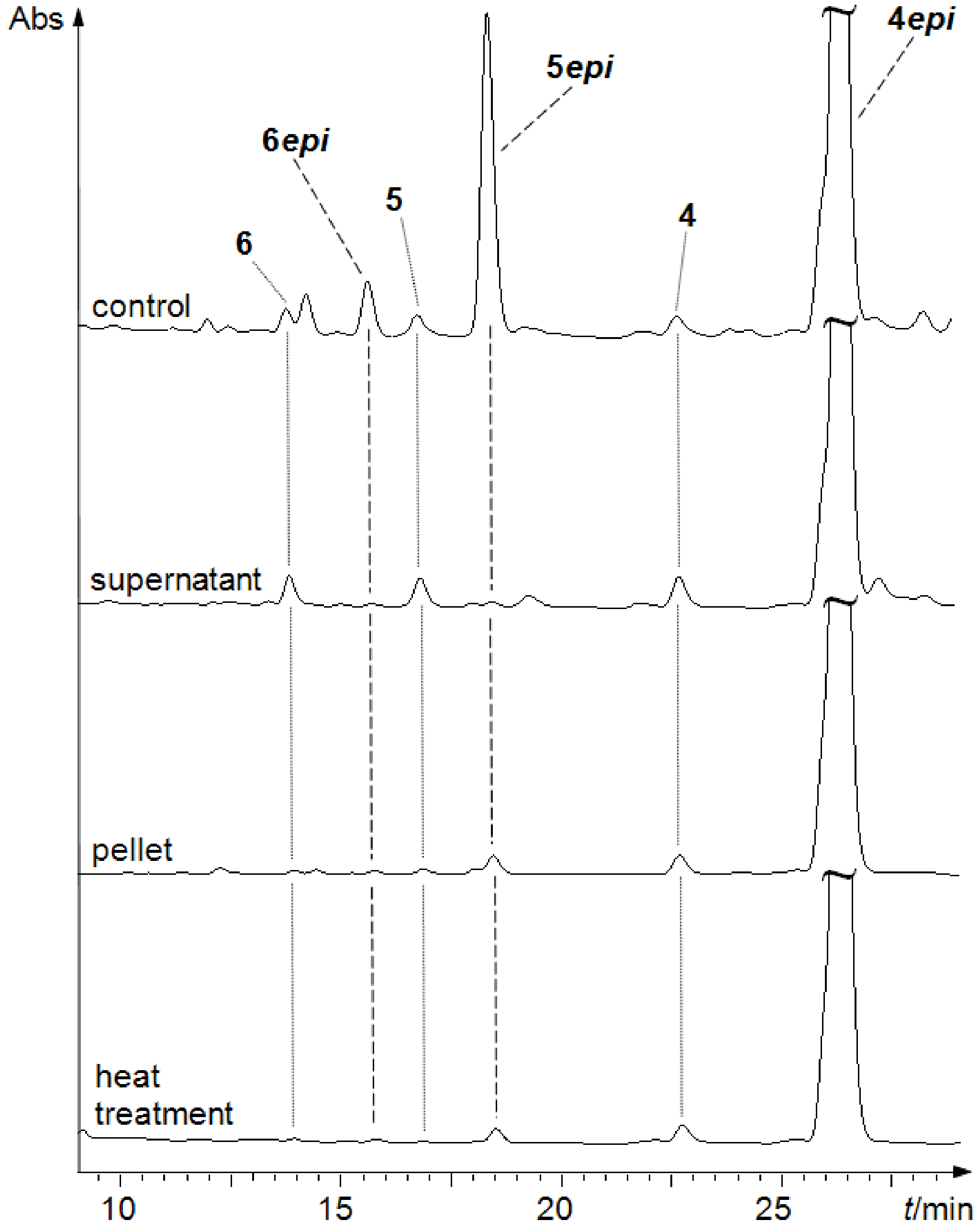

Figure S12: A homogenate of yellow senescent *Sp. wallisii* leaf parts was mixed with a solution of ***4epi*** in MeOH and was kept in the dark at room temperature for 30 minutes. HPLC analysis of the resulting extract displayed all expected catabolites (top, detection at 320 nm). Centrifugation of an *Sw* homogenate followed by separate addition of the ***4epi*** containing solution, either to the supernatant or to the pellet, furnished almost no oxidized derivatives of NCC ***4epi***. The same result was achieved when the plant material was heated up to 70°C for 10 minutes before addition of NCC ***4epi*** (bottom).

References

[1] C. Curty and N. Engel, *Phytochem.* **1996**, *42*, 1531-1536.
[2] S. Moser, M. Ulrich, T. Müller and B. Kräutler, *Photochem. Photobiol. Sci.* **2008**, *7*, 1577-1581.
[3] a) R. R. Ernst, G. Bodenhausen and A. Wokaun, *Principles of Nuclear Magnetic Resonance in One & Two Dimensions*, Clarendon Press, Oxford, **1987**, p; b) H. Kessler, M. Gehrke and C. Griesinger, *Angew. Chem. Int. Ed.* **1988**, *27*, 490-536.
[4] H. E. Gottlieb, V. Kotlyar and A. Nudelman, *J. Org. Chem.* **1997**, *62*, 7512-7515.
[5] a) J. B. Fenn, M. Mann, C. K. Meng, S. F. Wong and C. M. Whitehouse, *Science* **1989**, *246*, 64-71; b) T. Müller, S. Vergeiner and B. Kräutler, *Int. J. Mass Spectrom.* **2014**, *365-366*, 48-55.
